# In silico rationalisation of selectivity and reactivity in Pd-catalysed C–H activation reactions

**DOI:** 10.3762/bjoc.16.122

**Published:** 2020-06-25

**Authors:** Liwei Cao, Mikhail Kabeshov, Steven V Ley, Alexei A Lapkin

**Affiliations:** 1 Department of Chemical Engineering and Biotechnology, University of Cambridge, Cambridge CB3 0AS, UK; 2 Cambridge Centre for Advanced Research and Education in Singapore, CARES Ltd., CREATE Way, CREATE Tower #05-05, 138602 Singapore; 3 Department of Chemistry, University of Cambridge, Lensfield Rd, Cambridge CB2 1EW, UK; 4 Benevolent AI, Minerva Building, Babraham Research Campus, Cambridge CB22 3AT, UK

**Keywords:** C–H activation, density functional theory, reaction prediction

## Abstract

A computational approach has been developed to automatically generate and analyse the structures of the intermediates of palladium-catalysed carbon–hydrogen (C–H) activation reactions as well as to predict the final products. Implemented as a high-performance computing cluster tool, it has been shown to correctly choose the mechanism and rationalise regioselectivity of chosen examples from open literature reports. The developed methodology is capable of predicting reactivity of various substrates by differentiation between two major mechanisms – proton abstraction and electrophilic aromatic substitution. An attempt has been made to predict new C–H activation reactions. This methodology can also be used for the automated reaction planning, as well as a starting point for microkinetic modelling.

## Introduction

Periodically, our knowledge of chemistry is enriched with new transformations that provide significant breakthroughs by enabling new synthetic strategies. Such examples in recent years include olefin metathesis [1] as well as C–C and C–N coupling reactions [2], among the most obvious examples. While these reactions undoubtedly had very significant impacts on the development of much cleaner and efficient chemical synthesis strategies, the early days of all new transformations are invariably challenging, with very slow and protracted paths from the initial discoveries to the demonstrations of broad substrate applicability and robustness, that are expected of industrial catalytic processes. Today, there exist a number of fairly recently (re)discovered transformations, that are of potential high industrial significance, and where one can observe the same problem of a lack of robustness. Thus, any approach that may speed-up the transition from a discovery of a new transformation to it becoming a robust synthetic strategy, is highly desired.

Recent years have seen the emergence of new methods of research in chemistry and process development, which include high-throughput experiments [3], autonomous self-optimising reactors [4–6], as well as predictions of reaction outcomes and of reaction conditions based on machine learning (ML) and artificial intelligence (AI) tools [7–9]. Especially the methods of ML/AI for prediction of reaction outcomes are attracting a lot of attention. Prediction accuracies in the order of 70–80% for the reaction outcomes [9], and around 60–70% for reaction conditions [10], were recently demonstrated. While machine learning methods are showing great promise and continue to be improved upon, it is also clear that a ML model is unlikely to ever be able to compete in accuracy and interpretability with fully predictive mechanistic models, were it not for the prohibitively high cost of developing the mechanistic models based on accurate quantum chemical methods, such as the density functional theory (DFT) methods, decreases. Automation of DFT, as well as using results of DFT to develop less expensive predictive models, are the two approaches that may offer the alternatives to the fully data-driven statistical methods.

Here we demonstrate an approach that was developed to automate the DFT-level calculations of energies of the auto-generated reaction intermediates. These results were further used to generalize mechanistic knowledge of a class of reactions, and the developed models were used for in silico prediction of reaction outcomes. This approach was tested on the for green chemistry important class of C–H activation reactions. Whilst this study does not completely solve the problem of developing a robust chemical reaction, it offers an approach that is complementary to efforts of developing machine learning models for predicting reaction outcomes.

C–H activation reactions allow conversion of relatively inexpensive and abundant hydrocarbons into the more sophisticated value-added molecules [11]. With the notion of step-economical and environmentally friendly synthesis, direct functionalization of C–H bonds is a powerful strategy for the synthesis and derivatization of organic molecules [12]. Homogeneous catalysis employing transition metal complexes has been widely accepted as one of the most efficient ways to perform C–H activation-based synthesis with high selectivity under relatively mild conditions [13]. In particular, reactions involving palladium-catalysed activation of sp^2^ or sp^3^ C–H bonds of arenes or alkanes have been extensively investigated due to their wide scope and functional group tolerance [14].

A number of different mechanisms are proposed in the literature, explaining the experimental observations for C–H activation reactions, depending on the nature of a ligand (L_n_) and transition metal (M) in the catalytically active species (L_n_M). These mechanisms include four elementary steps: oxidative addition, σ-bond metathesis, electrophilic substitution and 1,2-addition, respectively [15]. Even though the mechanisms are inherently different, three most important aspects should be primarily taken into account when classifying and rationalising C–H activation reactions:

1. the proximity of C–H bond to the transition metal;
2. the energy of C–H bond cleavage within the transition metal coordination sphere;
3. the energy of a new M–C bond formed and the thermodynamic stability of organometallic product.

With new developments in computational chemistry, mechanistic studies using density functional theory (DFT) provide valuable insights into the reactivity of organometallic complexes in C–H activation reactions. Along with the huge increase in computing power, this method becomes practically feasible to build model systems that provide parameters of the actual experimental systems with acceptable accuracy [16]. Recently, a predictive tool using quantum mechanics descriptors was proposed for classifying whether the carbon atoms are active or inactive toward electrophilic aromatic substitution [17]. Also, a quantum mechanical approach was introduced to compute *ortho*-directing groups (DGs) in palladium-catalysed aromatic C–H activation reactions [18]. However, there is a big challenge remaining which is to apply the computational analysis to a large number of mechanistically different transformations, both described and novel, in order to start generating accurate in silico reaction predictions. Here, we report an algorithm with high-performance computing (HPC) implementation, which has been developed to automatically generate and analyse the structures of the intermediates, and which allows prediction of the final products. The application of the developed methodology is in predicting reactivity for various substrates within a class of reactions. Using analysis of the computational data, a threshold to distinguish between two possible reaction mechanisms was established.

## Computational Methods

The NWChem, an open source software package, was used for the DFT calculations. It is easily scalable and designed to solve large scientific computational problems efficiently employing modern supercomputer clusters [19]. The structures were generated by the Python module developed in house and explained in detail elsewhere [20]. Electronic energies were evaluated using Becke’s three-parameter hybrid B3LYP functional, while the molecular orbitals are expanded in triple-zeta all electron 6-31 set with added polarization and diffuse functions [6-31g(d,p)] [21]. B3LYP functional was proven to give accurate description of geometries, frequencies, relative stabilities of different conformers and the energy profile calculation [22]. Implementation of the tools is available at GitHub: https://github.com/sustainable-processes/Pd-catalysed_C-H_activation_reaction_prediction.

## Results and Discussion

### Computational approach to rationalise reactivity in Pd-catalysed C–H bond activation reactions

**Figure 1:**
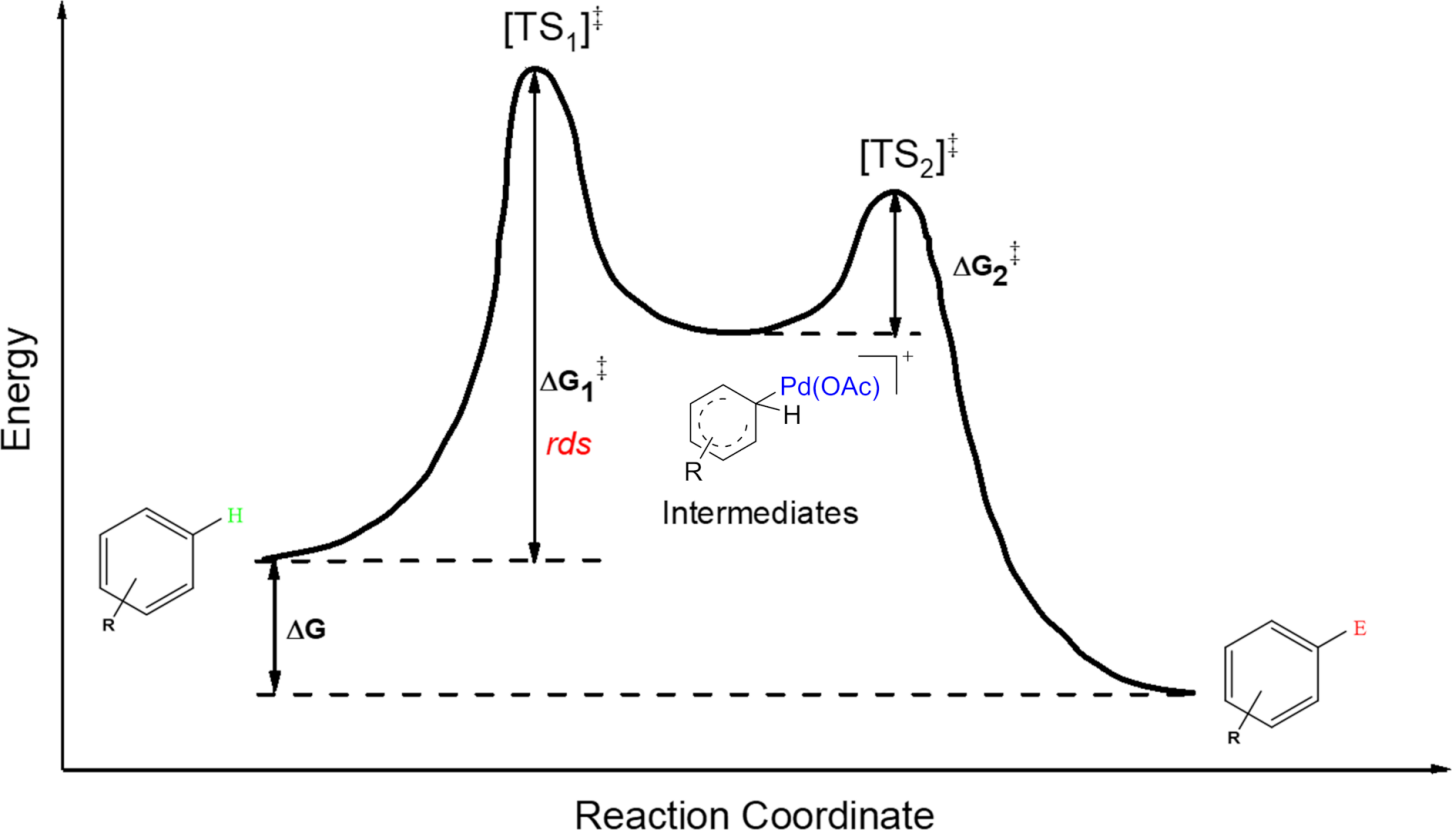
An approximate energy map for the electrophilic aromatic substitution mechanism.

Chemical reactivity is simultaneously influenced by many factors including catalysts, reactants, reaction conditions, and so on [23]. In order to achieve accurate and efficient reaction prediction, a mechanism-based method was chosen to direct quantum chemistry calculations and predictions, see Figure 1.

**Scheme 1:**
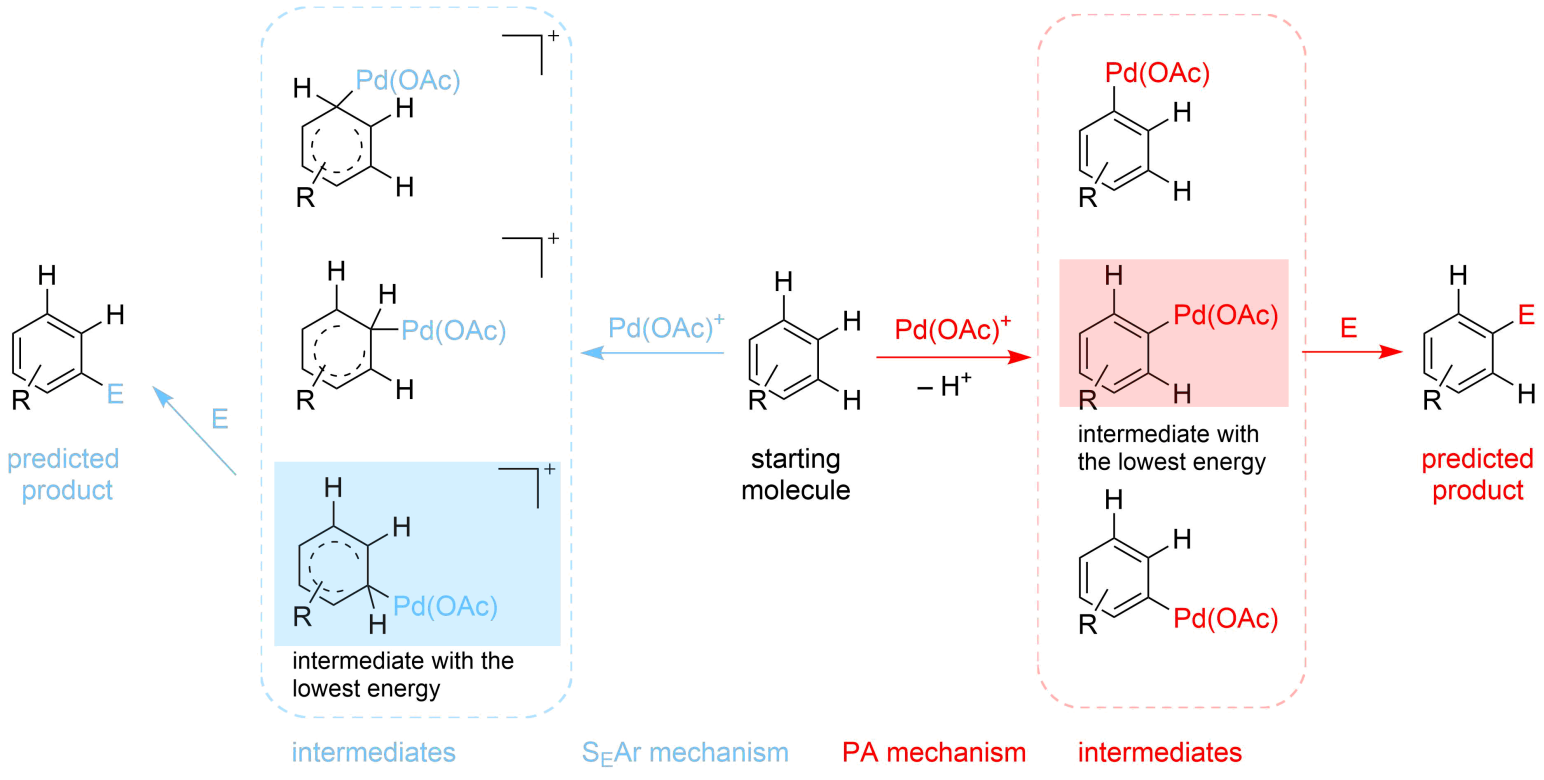
Schematic representation of the two mechanisms of Pd-catalysed C–H activation reaction considered in this study.

For the Pd(II)-catalysed C–H activation reactions, there are two main commonly accepted mechanisms: a) electrophilic aromatic substitution (S_E_Ar) mechanism and b) proton abstraction (PA) mechanism. The key step for the electrophilic aromatic substitution is an electrophilic attack by Pd(II) onto the aromatic substrate that also defines the regioselectivity of the overall process [24]. The key feature of the proton abstraction (PA) mechanism [25] is that the formation of the metal–carbon bond (M–C) occurs simultaneously with the cleavage of the carbon–hydrogen (C–H) bond, while the hydrogen is being transferred to a basic centre, Scheme 1.

Assuming the reaction proceeds through the formation of a relatively unstable intermediate (Figure 1) [26], the Hammond postulate can be applied to the electrophilic substitution reactions. The Hammond postulate states that a transition state will be structurally and energetically similar to the species (reactant, intermediate or product) nearest to it on the reaction path. In this case, the intermediates are likely to be close to, and resemble, transition states. Due to that, their relative energy of formation can be translated to relative reaction kinetic barriers and thus be used, as the first approximation, to predict distributions of the final products, as well as the relative reactivity of the substrates [27]. For the PA mechanism, it has not been shown that the Hammond postulate can also be employed. Nevertheless, it is still reasonable to propose that the Hammond postulate can similarly be applied as a first approximation to produce in silico predictions.

Employing the Python module [20] and OpenBabel executables [28], the 3D structures of the most stable conformers were generated from the 2D structure of a substrate. Subsequently, structures of all possible palladium intermediates representing both mechanisms (PA and S_E_Ar) were built for each conformer. A quick geometry optimization (maximum number of iteration steps was set to 5) was then applied to refine the intermediates and discard the ones with high energy (energy cut off of 10 kcal·mol^−1^). Full geometry optimisation followed by the frequency and thermochemistry analysis was then performed for the selected intermediates to obtain electronic energies. Multiple error handlers were implemented in order to automatically reprocess computational analysis for the intermediates when initial geometry optimisation failed. These include: (i) erroneous optimisation to a saddle point where the final structure is changed by applying a move along imaginary coordinate followed by standard geometry optimisation, (ii) failed optimisation due to the need of updating Hessian in cases where significant geometry change occurred – standard resubmission starting from the last coordinate, (iii) failure to perform initial guess due to particularly bad initial geometry – discard the conformer/intermediate, (iv) decomposed intermediate (no Pd–C bond determined by interatomic distance analysis) – discard intermediate.

### Literature validation

In order to test the developed algorithm, a representative literature data selection of Pd-catalysed C–H activation reactions, consisting of reactant, reagents, and product structures as well as reaction conditions, was taken and analysed. Thus, twelve substrates shown in Table 1 were submitted to the algorithm, assuming that both mechanisms are possible. Using the relative energies of the intermediates obtained, the theoretically expected regioselectivity of the selected reactions was devised and then compared against the previously reported experimental data.

**Table 1:** Comparison of the published experimental results with the computational predictions for the Pd(OAc)_2_-catalysed reactions.^a^

No [ref]	Starting molecule	Exp. cond.	Predicted active centre		Experimentally isolated product
			Via acidity mechanism	Via electrophilic mechanism	

1 [29]	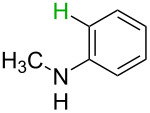	CO, EtOH, Pd(OAc)_2_, Cu(OAc)_2_, KOAc, DMF, KI, 100 °C, 13 h	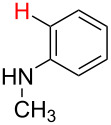	no stable intermediate	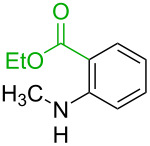
2 [30]	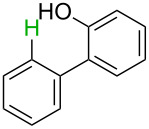	CO, Pd(OAc)_2_, Cu(OAc)_2_, PivOH, mesitylene, 120 °C, 6 h	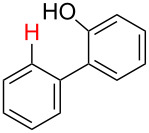	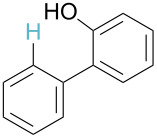	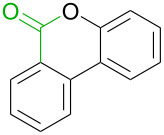
3 [31]	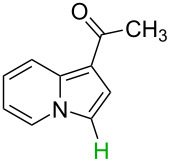	Cu(OAc)_2_, Pd(OAc)_2_, K_2_CO_3_, DMF, 60 °C, 0.6 h	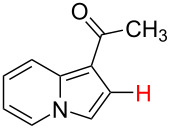	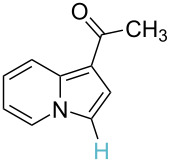	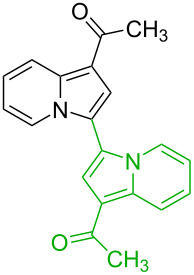
4 [32]	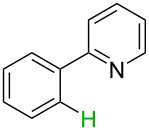	PhCOCO_2_H Pd(OAc)_2_, K_2_S_2_O_8_, MeCN, 25 °C, 16 h	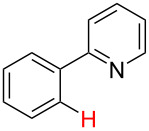	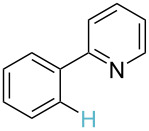	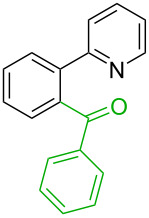
5 [33]	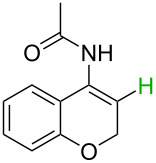	PhSi(OMe)_3_, Pd(OAc)_2_, AgF, dioxane, 80 °C, 16 h	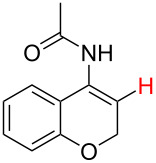	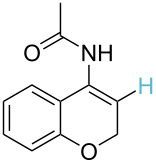	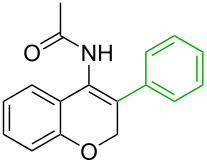
6 [32]	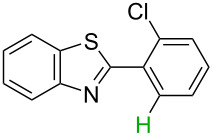	Ph-CHO, Pd(OAc)_2_, TBHP, toluene, 110 °C, 5 h	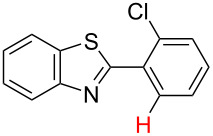	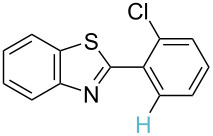	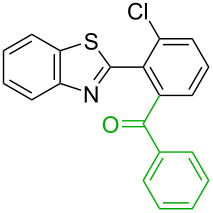
7 [34]	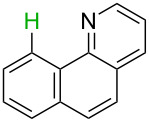	Ph-CHO, Pd(OAc)_2_, xylene, O_2_ 120 °C, 24 h	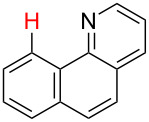	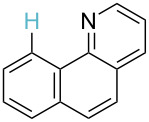	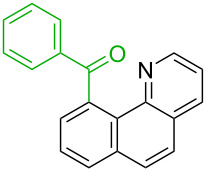
8 [35]	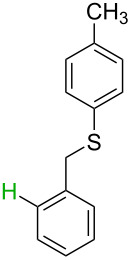	PhCOCO_2_H, Pd(OAc)_2_, Ag_2_CO_3_, DMF, 120 °C, 24 h	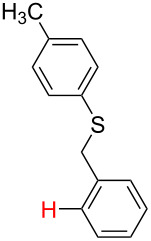	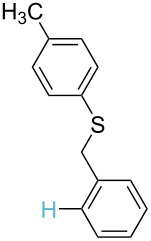	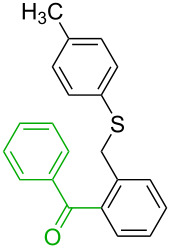
9 [36]	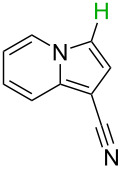	H-COOPh, Pd(OAc)_2_, I_2_, K_2_CO_3_, DMF, 100 °C, 12 h	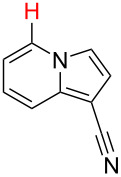	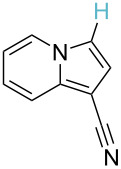	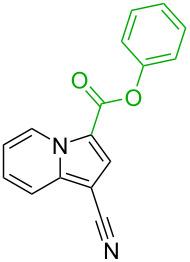
10 [37]	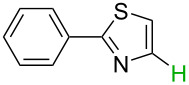	PhB(OH)_2_, Pd(OAc)_2_, TEMPO, phen, DMAc, O_2_, 100 °C, 48 h	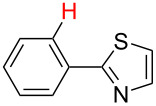	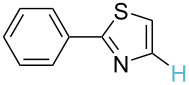	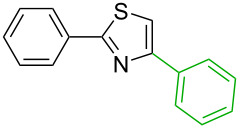
11 [38]	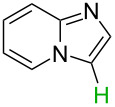	benzene, Pd(OAc)_2_, O_2_, HOAc, DMA, 130 °C, 20 h	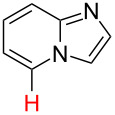	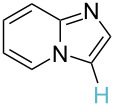	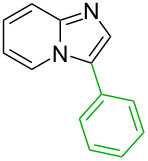
12 [39]	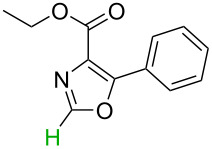	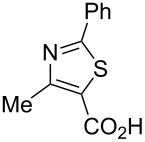 Pd(OAc)_2_, CuCO_3_, dioxane, DMSO, 140 °C, 16 h	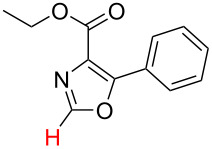	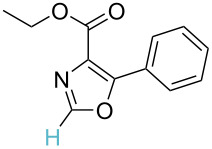	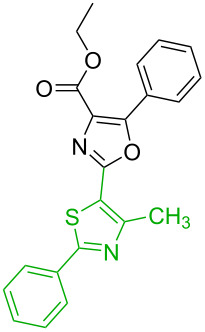

^a^Protons marked green are those that react under the conditions reported in the literature. Protons marked red and blue are the predicted active centres via the acidity and the electrophilic mechanisms, respectively.

For all the examples regioselectivity predicted by at least one mechanism matched the previously reported experimental results, see Table 1. In the cases where only one product was predicted it is expected to be isolated in high yield without the need of further purification from any other regioisomer. For the examples where formation of multiple products was expected due to the close energies of the respective reaction intermediates, the ratio of products was calculated from the relative energies of these intermediates using the Boltzmann distribution equation.

### Establishing the threshold between the two mechanisms

Although both, the proton-abstraction and the electrophilic aromatic-substitution, mechanisms are well established and described in the literature, it is not trivial to suggest the preferred mechanism for a given substrate based on a simple computational analysis. Through analysis of the results described above, the two-step evaluation algorithm was suggested.

Firstly, the optimised geometries were manually examined to ensure they represent the intermediates according to the particular mechanism. In particular, the bond length between the palladium atom and the corresponding carbon atom was given a maximum value of 2.4 Å to filter out inappropriate intermediates where there is no stable Pd–C bond [40].

Secondly, among the intermediates refined at the previous step, their relative Gibbs energies can be used to set a threshold establishing the likeliness of electrophilic aromatic substitution mechanism for C–H activation of a particular substrate. The more stable the ipso-complex between palladium acetate and the substrate is, the more likely the substrate is to follow the electrophilic mechanism. After performing the computational analysis of 12 examples which include five structures following the electrophilic mechanism, a threshold has been developed by choosing the example 6 as the reference, Table 1, and introducing the ipso-complex stability parameter. We define this parameter to be the energy difference between the most stable intermediate of the S_E_Ar mechanism and the one of the PA mechanism.

By comparing the computational results obtained to the literature experimental data, the two mechanisms can be segregated based on the following rules:

1. If the relative stability is below zero, the starting molecule will follow the proton abstraction mechanism.
2. If the relative stability is above five, the starting molecule will follow the electrophilic aromatic substitution mechanism.
3. If the relative stability is between zero and five, both mechanisms are regarded as plausible.

Although the rules set above seem rather approximate, they are consistent with the given examples, and further work aimed at increasing the accuracy and the scope of the algorithm is on-going. Based on the suggested rules, the predicted reactive centres for eight commercially available aromatic and heteroaromatic substrates as well as the most likely mechanisms are shown in Table 2.

**Table 2:** Predicting C–H activation bond for heteroaromatic compounds.^a^

No.	Starting molecule	Pred. mec.	Computational prediction			
			Acidity mechanism		Electrophilic mechanism	

1	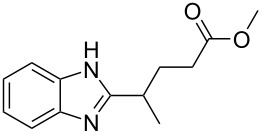	S_E_Ar	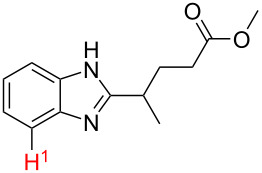	**H1:0.0**	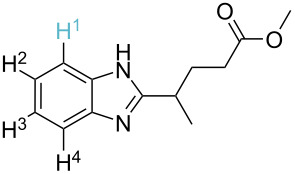	**H1:0.0**
2	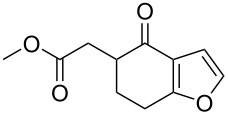	S_E_Ar	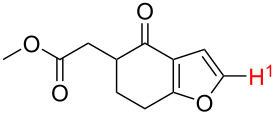	**H1:0.0**	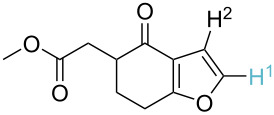	**H1:0.0** H2:0.5
3	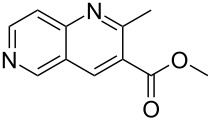	PA	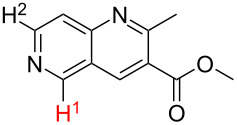	**H1:0.0** H2:0.7	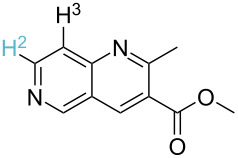	**H2:0.0** H3:0.9
4	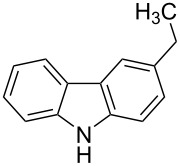	PA	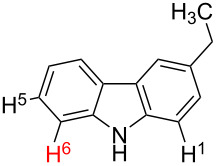	H1:1.9 H5:9.8 **H6:0.0**	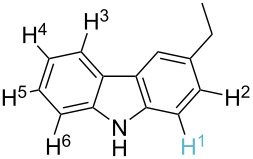	**H1:0.0** H2:3.9 H3:2.9 H4:2.9 H5:4.2
5	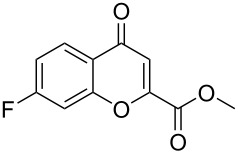	PA	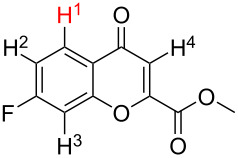	**H1:0.0** H2:2.7 H3:9.7 H4:6.2	no stable intermediate	
6	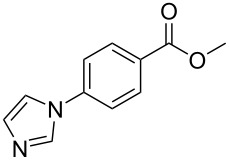	PA/S_E_Ar	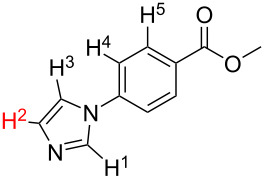	H1:0.2 **H2: 0.0** H3: 0.3 H4:0.9 H5:2.6	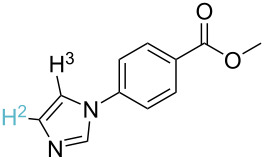	**H2:0.0** H3:2.7
7	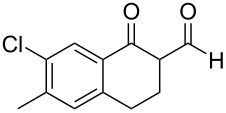	PA	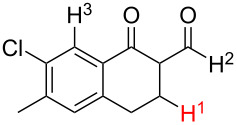	**H1:0.0** H2:2.8 H3:10.0	no stable intermediate	
8	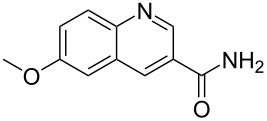	PA	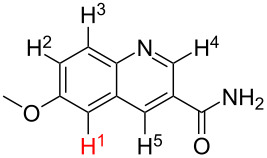	**H1:0.0** H2:0.8 H3:5.5 H4:4.3 H5:6.0	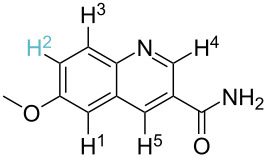	H1:0.1 **H2:0.0** H3:14.9 H4:15.0 H5:15.8

^a^Most probable intermediates for each mechanism are shown, and relative Gibbs free energy are given in kcal mol^–1^. If only one possible intermediate is given, it means that either the other intermediates are unstable or the other intermediates have 10 more kcal mol^−1^ Gibbs free energy than the most probable one. ‘no stable intermediate’ means instead of sitting on the corresponding carbon, the palladium sits on alternative atom. The predicted mechanism is given based on the threshold described in the previous section.

In order to test the algorithm and the value of the threshold, an additional set of six examples was analysed, and the results are shown in Table 3. Both selectivity and mechanism were correctly identified by the algorithm applying the previously set threshold to the SnAr intermediate stability (intermediates 5), which is shown in Table 4.

**Table 3:** A comparison of the published experimental results with the computational predictions for the Pd(OAc)_2_-catalysed reactions.^a^

No [ref]	Starting molecule	Exp. cond.	Predicted active center		Experimentally isolated product
			Via acidity mechanism	Via electrophilic mechanism	

1 [41]	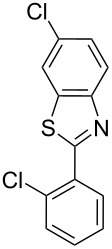	Pd(OAc)_2_, TBHP, toluene, 120 °C, 6 h	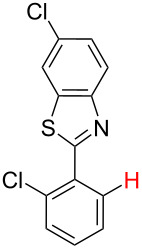	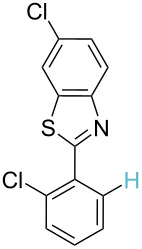	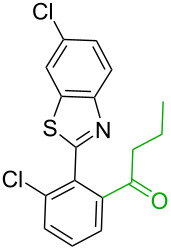
2 [42]	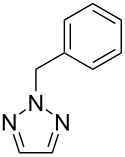	Pd(OAc)_2_, TBHP, DCE 80 °C, 16 h	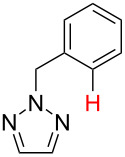	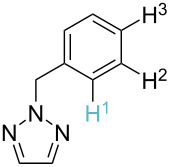 **H1:0.0** H2:26.0 H3:27.5	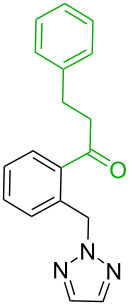
3 [43]	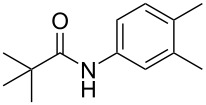	Pd(OAc)_2_, TBHP, toluene, TFA, 40 °C, 3 h	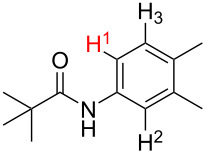 **H1:0.0** H2:1.9 H3:7.6	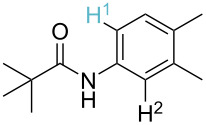 **H1:0.0** H2:3.2	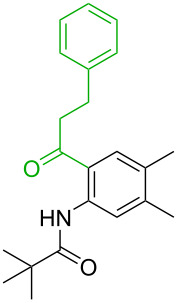
4 [44]	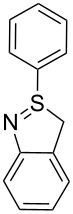	Pd(OAc)_2_, toluene, TBHP, 110 °C, 5 h	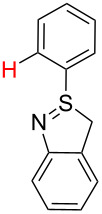	no stable intermediate	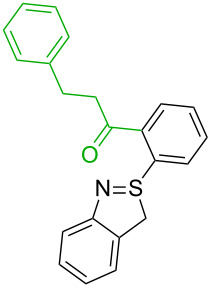
5 [45]	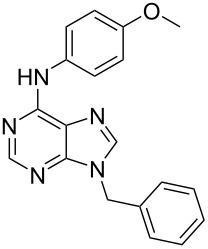	Pd(OAc)_2_, 1,4-dioxane, AcOH, DMSO, TBHP, 110 °C, 24 h	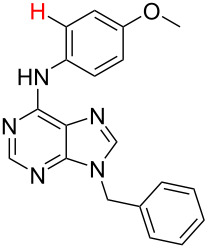	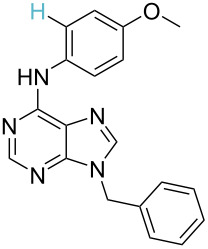	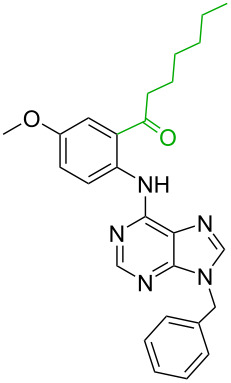
6 [46]	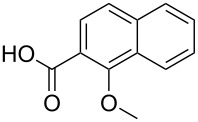	Ag_2_CO_3_, Pd(OAc)_2_, NaOAc, CO, 1,4-dioxane, 130 °C, 18 h	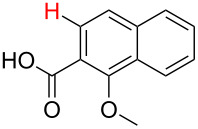	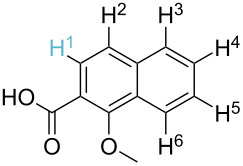 **H1:0.0** H2:2.6 H3: 2.2 H4:2.5 H5:2.2 H6:2.8	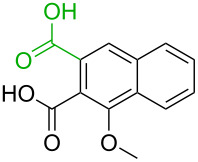

^a^Protons marked red and blue are the predicted active centres via the acidity and the electrophilic mechanisms, respectively.

**Table 4:** A mechanism threshold tested based on the literature examples.^a^

Entry	Gibbs free energy of Pd–substrate [Hartree]	d(Pd–C) [Å]	Relative stability	Predicted mechanism	Reported mechanism

1	−355.5652	2.3005	3.0777	PA/S_E_Ar	PA/S_E_Ar
2	−355.5577	2.3778	−1.6369	PA	PA
3	−355.5626	2.1345	1.4558	PA S_E_Ar	PA/S_E_Ar
4	no stable intermediate	–	–	PA	PA
5	355.5717	7.1781	2.2326	S_E_Ar	S_E_Ar
6	−355.5254	2.1680	−21.9298	PA	PA

^a^Gibbs free energy of Pd-substrate is obtained by calculating the Gibbs free energy difference between starting molecule and the most probable intermediate in Hartree. The distance between the palladium atom and the corresponding carbon are measured based on the web-based molecular structure virtualization, which can be accessed through https://leyscigateway.ch.cam.ac.uk/index.php.

## Conclusion

A computational algorithm rationalising the existing palladium catalysed C–H activation reactions has been developed. Computational threshold to distinguish between the two main mechanisms, proton abstraction (PA) and electrophilic aromatic substitution (S_E_Ar) mechanism, has been proposed and tested against literature experimental data. This model can give not only the most probable reactive site and the appropriate mechanism, but also provides information for further kinetic studies and process development, thus contributing to the development of robust new chemical transformations.

## Supporting Information

File 1 Comutational details, comparison of data, mechanistic threshold, Cartesian coordinates and energies.
