# Supplementary material for: In silico rationalisation of selectivity and reactivity in Pd-catalysed C–H activation reactions
Source: Beilstein J Org Chem. 2020 Jun 25;16:1465–75. doi: 10.3762/bjoc.16.122 (PMC7323619; doi:10.3762/bjoc.16.122)
Supplement: File 1 — Comutational details, comparison of data, mechanistic threshold, Cartesian coordinates and energies. [file Beilstein_J_Org_Chem-16-1465-s001.pdf]

BEILSTEIN JOURNAL OF ORGANIC CHEMISTRY

Supporting Information

for

In silico rationalisation of selectivity and reactivity in Pd-catalysed C–H activation reactions

Liwei Cao, Mikhail Kabeshov, Steven V. Ley and Alexei A. Lapkin

Beilstein J. Org. Chem. **2020,** *16,* 1465–1475. doi:10.3762/bjoc.16.122

Comutational details, comparison of data, mechanistic threshold, Cartesian coordinates and energies

License and Terms: This is a supporting information file under the terms of the Creative Commons Attribution License (http://creativecommons.org/licenses/by/4.0). Please note that the reuse, redistribution and reproduction in particular requires that the authors and source are credited.
The license is subject to the *Beilstein Journal of Organic Chemistry* terms and conditions: (https://www.beilstein-journals.org/bjoc)

Table of Contents

1. Computational approach to C–H reactivity analysis and prediction
2. Comparison of the published experimental results with the computational predictions for the $Pd(OAc)_2$-catalysed reactions
3. A mechanism threshold
4. Cartesian coordinates, uncorrected electronic energies and screenshots of 3D representations of computed structures

1. Computational approach to C–H reactivity analysis and prediction

Chemical reactivity is simultaneously influenced by many factors including catalyst, reactants, reaction conditions, etc.[10] The key idea is to achieve an accurate as well as efficient reaction prediction, and our approach is based on organic chemistry mechanism rules and molecular modelling, which leads to a mechanism-based method. In the following section, the technical details of how to achieve the result in the main article is explained and illustrated.

1.1 Computational methods

The software packages used in this research project include Python 2.7.9 for reaction prediction algorithm; Chemcraft, MarvinSketch (64bit), MarvinSpace (64bit) and OpenBabel 2.3.2 for starting molecular structure and its conformation generating; Gaussian 09 and NWChem 6.6 for DFT calculation.

All the computational calculations were performed on either the Sustainable Reaction Engineering Group' machine *Gtuhanai* with 1 node containing 16 Intel® Xeon® E5-2650 cores (@2.60 GHz, 64GB RAM per node), or the Cambridge High Performance Computing Cluster *Darwin* with 600 nodes each containing 16 Intel® Xeon® E5-2670 cores (@2.60GHz, 64GB RAM per node).

1.2 Intermediates generation based on different mechanisms

Within this algorithm, intermediates for different mechanisms will be automatically generated. At first, the structure of starting molecules will be drawn in MarvinSketch (64bit) software and will be saved as a xyz file. Also, with OpenBabel 2.3.2, an open source chemistry toolbox, the xyz format file will be converted into different file formats for further calculation: smiles format, mol2 format, sdf format as well as conf format which contains conformations of the starting structure. Next, the program will only pick five conformers of the starting molecule with lowest Gibbs free energy and save it as input for the follow-up calculation. And then, depending on mechanisms, the starting structures will go through different paths within this program to generate possible intermediates. The workflow is illustrated in the scheme below.

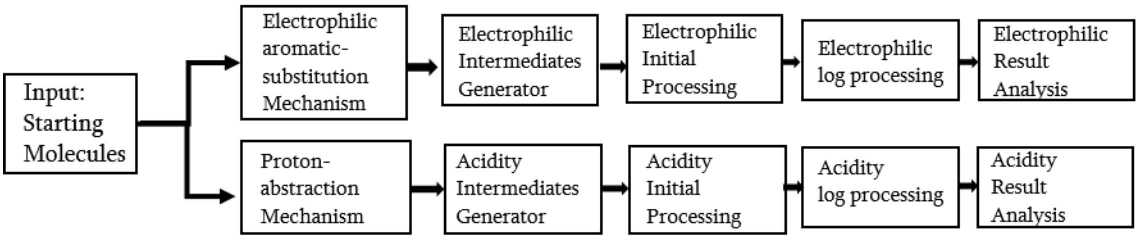

Scheme S1. Workflow for the computational algorithm.

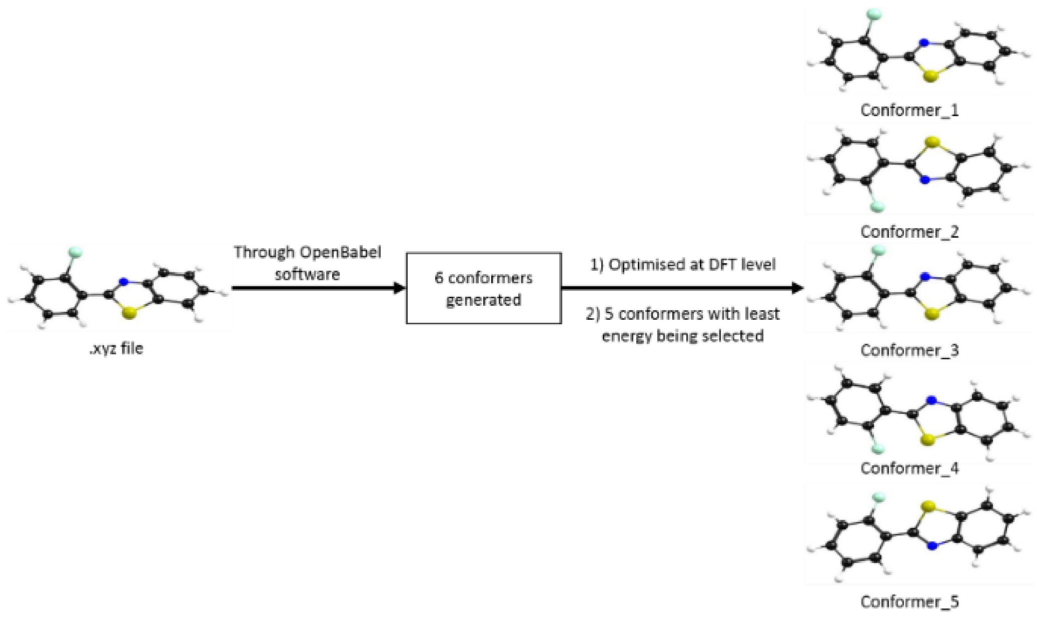

Scheme S2. Illustration of starting structure automatic conformer generation.

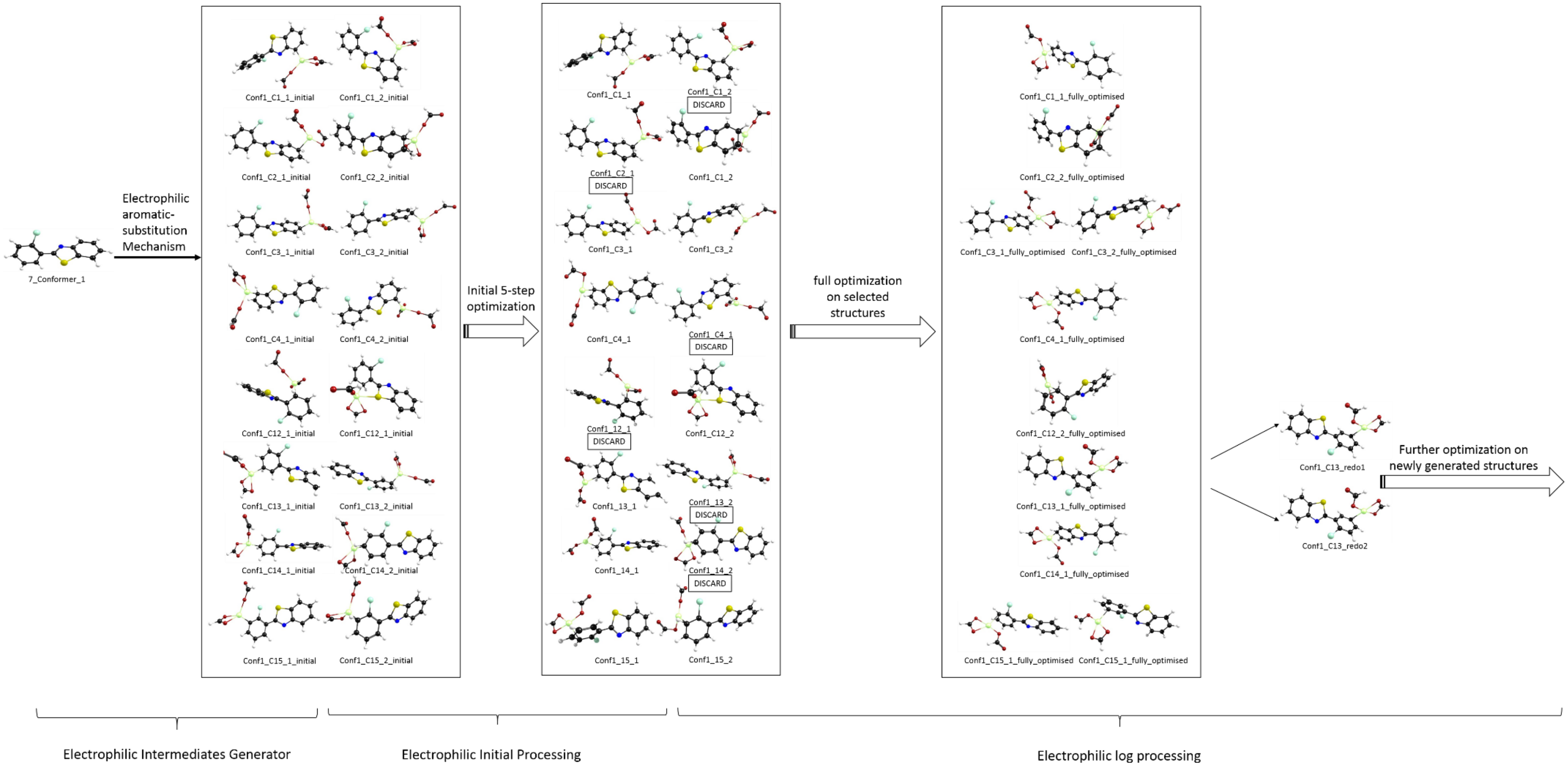

Scheme S3. Illustration of intermediates generation of the S$_E$Ar mechanism and the following optimization on DFT level procedures.

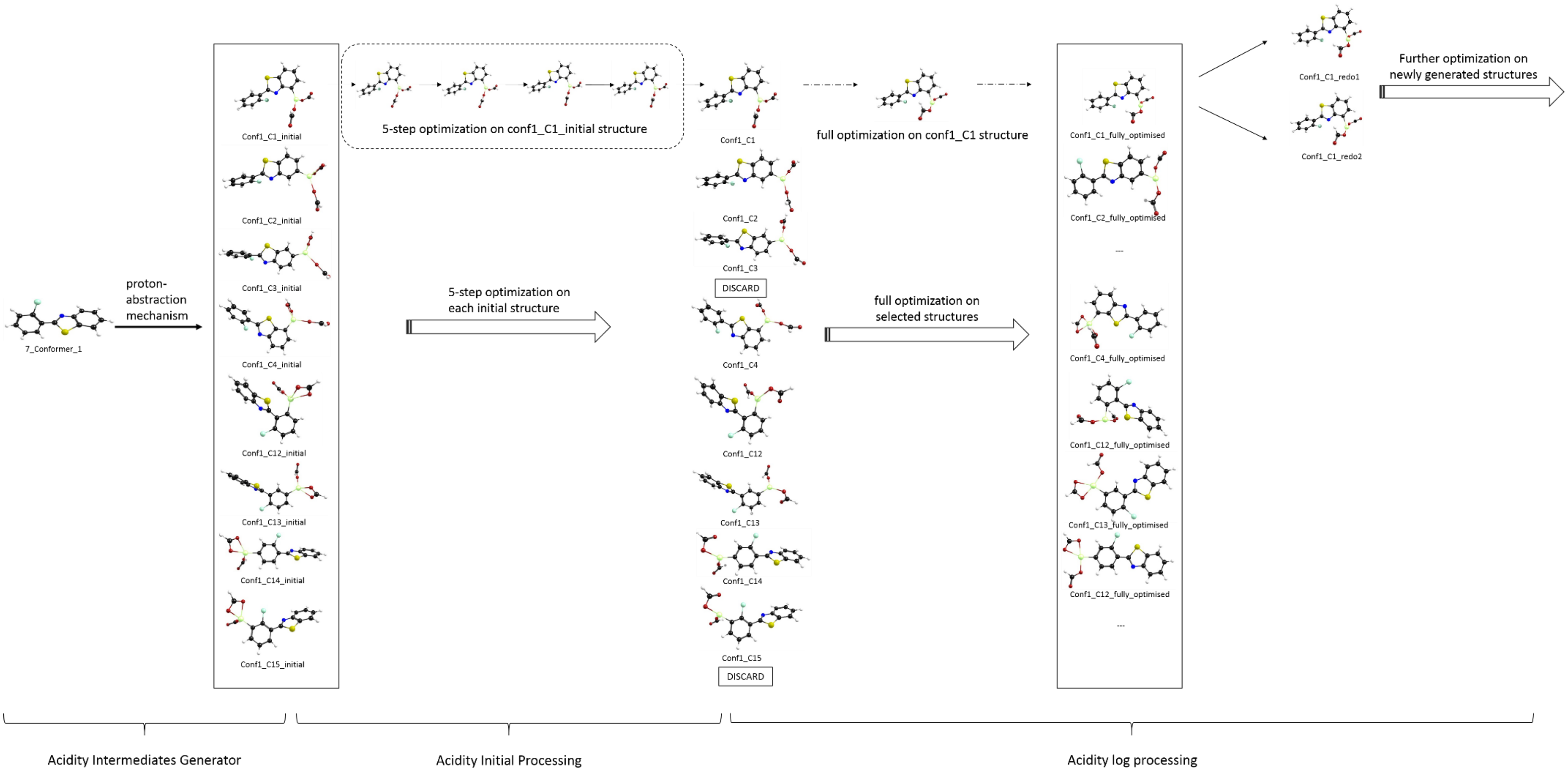

Scheme S4. Illustration of intermediates generation of the PA mechanism and the following optimization on DFT level procedures.

1.3 Exchange and correlation functionals evaluation for DFT calculation

As mentioned in the previous chapter, varying functionals and basis sets will cause changes in calculation of thermodynamic properties of molecules. In order to choose functionals suitable for transition metal catalysed intermediates calculation, palladium(II)-catalysed intermediates in particular, a crosscheck for different functionals need to be conducted.

Four different exchange and correlation functionals have been chosen for testing, respectively wB97xD, B3LYP, TPSSh and M06-2X (Table1). Ten examples from Reaxys database were chose to check exchange and correlation functionals for the calculation at density functional theory level. For those ten examples[1], starting molecules, possible intermediates and final product were calculated with the four different functionals mentioned above.

Table S1. Introduction for four different exchange and correlation functionals

Functionals	Description
wB97xD[i]	The latest functional from Head-Gordon and co-workers, which includes the version of Grimme's D2 dispersion.
B3LYP[ii]	The hybrid functional using Becke 3 parameters and the Lee-Yang-Parr functional.
TPSSh[iii]	The hybrid functional using the TPSS functionals (The exchange functional of Tao, Perdew, Staroverov, and Scuseria, 2003).
M06-2X[iv]	The hybrid functional of Truhlar and Zhao (2008).

These four functionals were implemented for DFT calculation, and then results were compared. As can be seen in Table 2, in this palladium catalysed system, different exchange and correlation functionals only cause minor influence to the results. In this case, the most popular and well-developed functional B3LYP functional[v] was selected for further calculation in this project.

Table S2. Result for functionals evaluation of example_6[50g]. Uncorrected Gibbs free energy (E), corrected Gibbs energy (G) and enthalpy (H) were calculated based on different exchange and correlation functionals.

		Starting molecule	Intermediate 1	Intermediate 2
		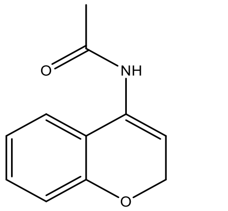	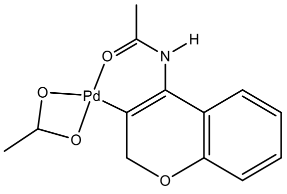	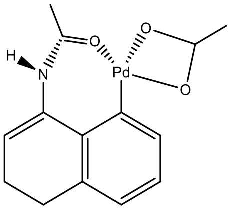
Thermal correction to enthalpy		0.2154	0.2644	0.2620
Thermal correction to Gibbs free energy		0.1641	0.1985	0.1938
WB97xD	E	-631.0114	-986.6362	-986.5765
	H	-630.7960	-986.3718	-986.3146
	G	-630.8473	-986.4377	-986.3828

[1] Examples selected here consistent with Table 3.

B3LYP	E	-631.1776	-987.0960	-987.0395
	H	-630.9622	-986.8317	-986.7775
	G	-631.0135	-986.8975	-986.8457
TPSSh	E	-631.2561	-987.1196	-987.0540
	H	-631.0407	-986.8552	-986.7920
	G	-631.0920	-986.9211	-986.8602
M06-2X	E	-630.9771	-986.6908	-986.6414
	H	-630.7616	-986.4264	-986.3794
	G	-630.8130	-986.4923	-986.4476

1.4 Geometry optimization and electronic structure determination at DFT level

Once the possible intermediates were generated based on the different mechanisms, a two-step optimization procedure was carried out in order to balance the computational time and the accuracy of the results.

At first, geometry of all generated intermediates was optimized over 5 iterations[2]. Then the structures having higher energy by a value of 10 kcal·mol^{-1} as compared to the least energy intermediate were crossed out, the remaining structure were then fully optimized and thermochemistry applied[3]. The geometry of the structures was considered stable and correct only if no negative vibrational frequencies were found after calculating force constants and vibrational frequencies. Moreover, for the unstable intermediates, the algorithm would automatically generate[4] another structure based on the former calculation as a starting point for further optimization.

All calculations were performed with the open source NWChem software package[vi] by using Becke's three-parameter hybrid B3LYP functional, while the molecular orbitals are expanded in triple-zeta all electron 6-31 set with added polarization and diffuse functions [6-31g(d,p)].[vii] B3LYP functional has been proven to give accurate description of geometries, frequencies, relative stabilities of different conformers and the energy profile calculation, not only in previous literatures,[viii] but also in the benchmarking studies done previously using Gaussian 09 software.

1.5 Selectivity analysis

Within the results analysis step, a text parsing and geometry operation module, coded in Python, has been developed to read and analyse the data from NWChem plain text outputs. By comparing Gibbs free energies of each intermediates, most reactive site for the given structure can be predicted which corresponds to the most stable intermediates. Moreover, the expected regioselectivity can be calculated based on the relative energy of intermediates using the Boltzmann distribution equation $ratio = e^{-\Delta E/RT}$.[48]

[2] As Initial Processing step shown in Scheme 3.

[3] As Log Process step shown in Scheme 3.

[4] The process of generating two new structure as starting point for further optimization is carried out within NWChem software.

2. Comparison of the published experimental results with the computational predictions for the $Pd(OAc)_2$-catalysed reactions

Table S3. Comparison of the published experimental results with the computational predictions for the $Pd(OAc)_2$-catalysed reactions.

Protons marked green are those that react under the conditions reported in the literature. Protons marked red and blue are the predicted active centers via the acidity and the electrophilic mechanisms respectively.

Entry [ref]	Starting molecule	Experimental conditions	Predicted active center				Experimentally isolated product
			Via acidity mechanism		*Via* electrophilic mechanism		
1 [29]	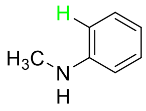	CO, EtOH, $Pd(OAc)_2$, $Cu(OAc)_2$, KOAc, DMF, KI, 100 °C, 13h	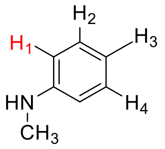	**H1:0.0**<br>H2:1.2<br>H3:0.4<br>H4:2.1	NO STABLE INTERMEDIATE		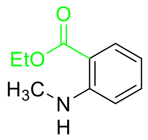
2 [30]	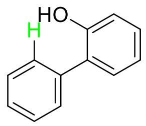	CO, $Pd(OAc)_2$, $Cu(OAc)_2$, PivOH, mesitylene, 120 °C, 6h	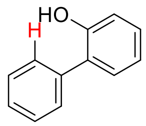		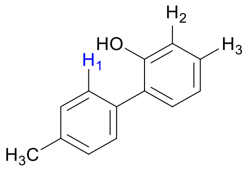	**H1:0.0**<br>H2:1.2<br>H3:2.0	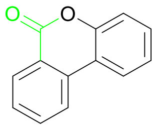
3 [31]	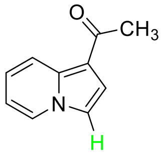	$Cu(OAc)_2$, $Pd(OAc)_2$, K_2CO_3, DMF, 60 °C, 0.6h	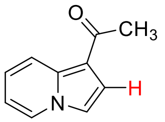		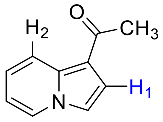	**H1:0.0**<br>H2:2.9	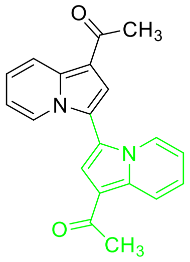

4 [32]	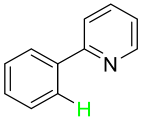	$PhCOCO_2H$ $Pd(OAc)_2$, $K_2S_2O_8$, MeCN, 25 °C, 16h	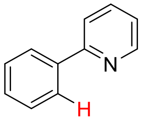		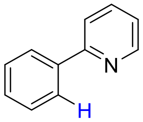		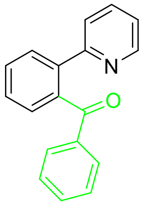
5 [33]	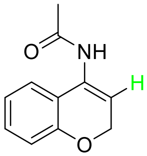	$PhSi(OMe)_3$, $Pd(OAc)_2$, AgF, dioxane, 80 °C, 16h	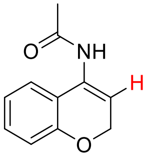		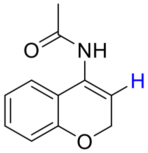		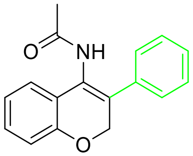
6 [32]	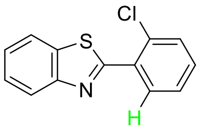	Ph-CHO, $Pd(OAc)_2$, TBHP, toluene, 110 °C, 5h	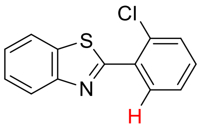		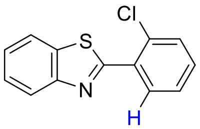		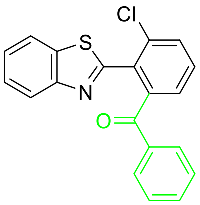
7 [34]	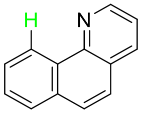	Ph-CHO, $Pd(OAc)_2$, Xylene, O_2 120 °C, 24h	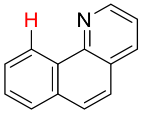		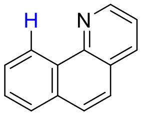		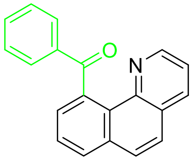
8 [35]	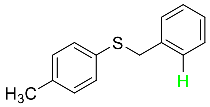	$PhCOCO_2H$, $Pd(OAc)_2$, Ag_2CO_3, DMF, 120 °C, 24h	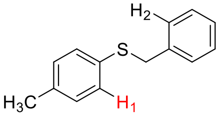	**H1: 0.0**<br>H2: 10.0	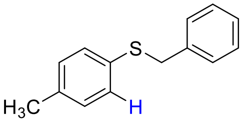		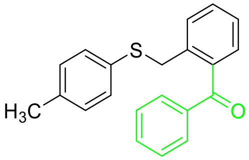

9 [36]	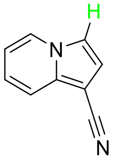	H-COOPh, Pd(OAc)$_2$, I_2, K_2CO_3, DMF, 100 °C, 12h	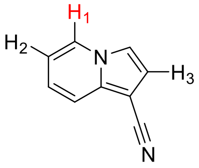	**H1:0.0** H2:1.3 H3:1.0	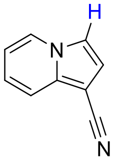		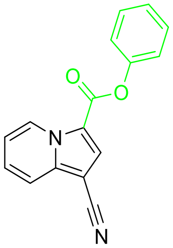
10 [37]	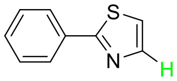	PhB(OH)$_2$, Pd(OAc)$_2$, TEMPO, phen, DMAc, O_2 100 °C, 48h	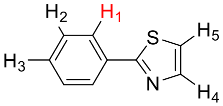	**H1:0.0** H2:13.8 H3:13.8 H4:14.0	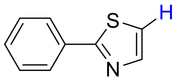	**H1:0.0** H2:5.8	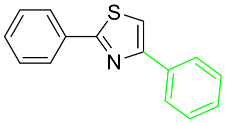
11 [38]	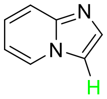	Benzene, Pd(OAc)$_2$, O_2, HOAc, DMA, 130 °C, 20h	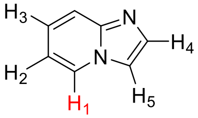	**H1:0.0** H2:16.4 H3:12.9 H4:8.2 H5:14.2	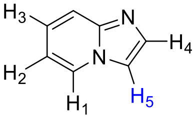	H1:8.4 H2:11.1 H3:10.2 H4:1.4 **H5:0.0**	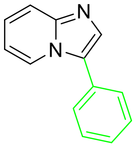
12 [39]	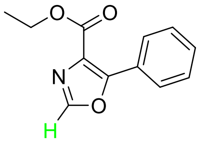	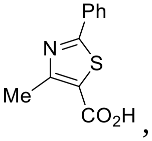 , Pd(OAc)$_2$, $CuCO_3$, dioxane, DMSO, 140 °C, 16h	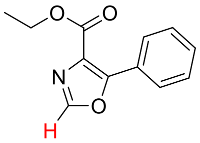		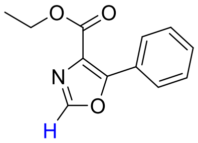		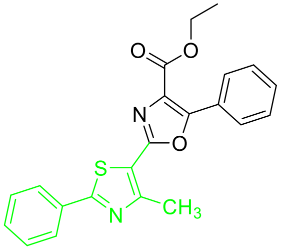

Table S4. Comparison of the published experimental results with the computational predictions for the $Pd(OAc)_2$-catalysed reactions.

Protons marked green are those that react under the conditions reported in the literature. Protons marked red and blue are the predicted active centers via the acidity and the electrophilic mechanisms respectively.

Entry [ref]	Starting molecule	Experimental conditions	Predicted active center				Experimentally isolated product
			Via acidity mechanism		*Via* electrophilic mechanism		
1 [ix]	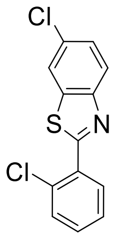	$Pd(OAc)_2$, TBHP, toluene, 120 °C, 6h	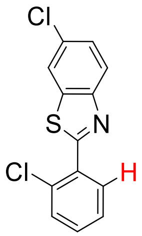		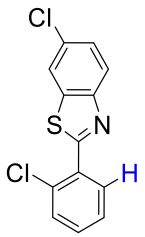		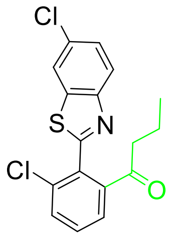
2 [x]	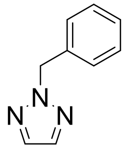	$Pd(OAc)_2$, TBHP, DCE 80 °C, 16h	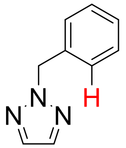		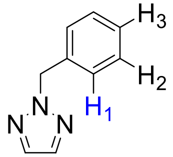	**H1:0.0** H2:26.0 H3:27.5	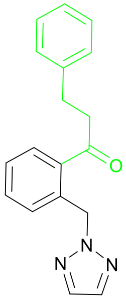
3 [xi]	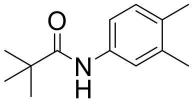	$Pd(OAc)_2$, TBHP, toluene, TFA, 40 °C, 3h	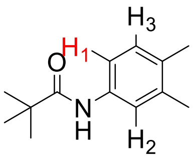	**H1:0.0** H2:1.9 H3:7.6	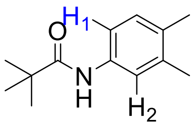	**H1:0.0** H2:3.2	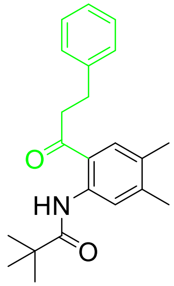

4 [xii]	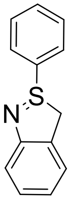	$Pd(OAc)_2$, Toluene, TBHP, 110°C, 5h	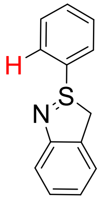		NO STABLE INTERMEDIATE		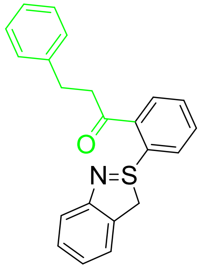
5 [xiii]	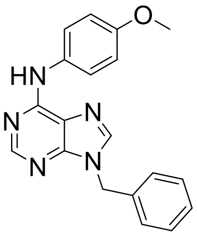	$Pd(OAc)_2$, Dioxane, AcOH, DMSO, TBHP, 110°C, 24h	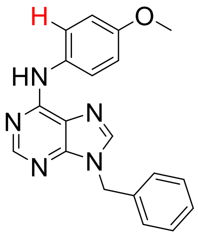		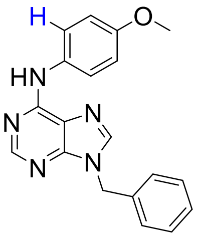		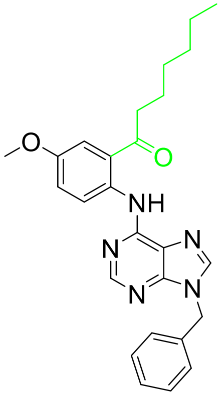
6 [xiv]	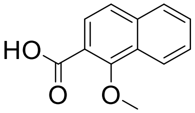	Ag_2CO_3, $Pd(OAc)_2$, NAoaC, CO, 1,4-dioxane, 130 °C, 18h	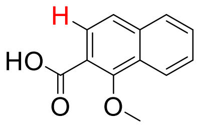		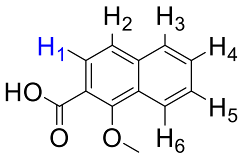	**H1:0.0**<br>H2:2.6<br>H3: 2.2<br>H4:2.5<br>H5:2.2<br>H6:2.8	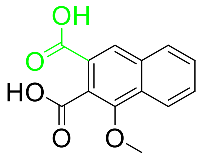

3. A mechanism threshold

3.1 A mechanism threshold calibrated based on literature examples

Entry	Gibbs free energy of Pd-substrate / Hartree	d(Pd-C) / Å	Relative stability	Predicted mechanism	Reported mechanism
1	No stable intermediate	-	-	PA	-
2	-355.5520	2.2246	-5.2968	PA	PA
3	-355.5715	2.1025	6.9666	S_EAr	S_EAr
4	-355.5547	2.3306	-3.5861	PA	PA
5	-355.5780	2.2049	11.0356	PA	PA
6	-355.5604	2.3097	0.0000	PA	PA
7	-355.5656	2.2591	3.2702	PA/ S_EAr	PA
8	-355.5506	2.2417	-6.1591	PA	PA
9	-355.5737	2.1199	8.3541	S_EAr	S_EAr
10	-355.5682	2.2210	4.8918	PA/ S_EAr	S_EAr
11	-355.5730	2.1240	7.9101	S_EAr	S_EAr
12	-355.5508	2.3204	-6.0532	PA	PA

3.2 A mechanism threshold tested on 6 literature examples

Entry	Gibbs free energy of Pd-substrate / Hartree	d(Pd-C) / Å	Relative stability	Predicted mechanism	Reported mechanism
1	-355.5652	2.3005	3.0777	PA/ S_EAr	PA
2	-355.5577	2.3778	-1.6369	PA	PA
3	-355.5626	2.1345	1.4558	PA/ S_EAr	PA
4	No stable intermediate	-	-	PA	PA
5	355.5717	7.1781	2.2326	S_EAr	S_EAr
6	-355.5254	2.1680	-21.9298	PA	PA

3.3 Using the mechanism threshold to predict on the new starting molecules

Entry	Gibbs free energy of Pd-substrate / Hartree	d(Pd-C) / Å	Relative stability	Predicted mechanism
1	-355.5795	2.2532	11.9884	S_EAr
2	-355.5740	2.2991	8.5297	S_EAr
3	-355.5480	2.1296	-7.7624	PA
4	-355.5560	2.2834	-2.7908	PA

5	No stable intermediate	-	-	PA
6	-355.5666	2.1634	3.8766	PA/ S_EAr
7	No stable intermediate	-	-	PA
8	-355.5435	2.2045	-10.5740	PA

4. Cartesian coordinates, uncorrected electronic energies and screenshots of 3D representations of computed structures

4.1 Result of literature validation

No.	Cartesian coordinates, energies			
1	C	0.366954790	-1.596401070	-0.061240220
	C	1.166616020	-2.758535870	-0.017844030
	C	2.543138750	-2.620606200	0.274682630
	C	3.098252890	-1.375077300	0.536224960
	C	2.301879980	-0.219050880	0.527116250
	C	0.953235060	-0.363618240	0.197152990
	N	0.638630670	-4.018673480	-0.239707770
	C	-0.706607760	-4.226199520	-0.736990990
	H	-0.686214900	-1.649225120	-0.312534290
	H	3.168101100	-3.509576950	0.312829830
	H	4.155627630	-1.299082950	0.776216000
	H	2.734246490	0.747058770	0.791467530
	H	1.310831560	-4.728832260	-0.480709910
	H	-0.870780860	-5.297593100	-0.872159200
	H	-0.898663200	-3.722448610	-1.697357090
	H	-1.447483410	-3.866028340	-0.014366360
	Pd	0.097337040	1.356618410	0.008617660
	O	-1.410419960	3.051925890	-0.178470820
	C	-2.285528910	2.153860510	-0.089467230
	C	-3.761145430	2.456820370	-0.144728650
	O	-1.930575960	0.918170190	0.053503980
	H	-3.919397150	3.530791940	-0.245647790
	H	-4.247480850	2.089663060	0.763606930
	H	-4.212677390	1.932592020	-0.992282600
	Total DFT energy = -682.060622205886			
	One electron energy = -2704.074397721121			
	Coulomb energy = 1243.576182775515			
	Exchange-Corr. energy = -89.789822198948			
	Nuclear repulsion energy = 868.227414938667			
2	C	2.798697960	-0.271975490	-1.311267610
	C	2.549897200	-0.197307060	-2.677020580

	C	1.244714540	-0.033915360	-3.159313290
	C	0.205363010	0.067992830	-2.224290870
	C	0.441228430	0.008853030	-0.848162990
	C	1.757810520	-0.185837070	-0.365710870
	C	1.335442750	0.318748170	2.071105260
	C	2.069040000	-0.345585090	1.077554940
	C	3.104669160	-1.181144170	1.533009390
	C	3.378683360	-1.333610560	2.891523520
	C	2.618137410	-0.656414970	3.846207820
	C	1.583411030	0.182045250	3.429436860
	C	0.965060120	0.058494090	-4.640504510
	O	0.262663990	1.169558610	1.699847390
	H	3.822507390	-0.384231730	-0.965048650
	H	3.379058100	-0.267256600	-3.376733450
	H	-0.817305980	0.175993880	-2.576209260
	H	3.680024640	-1.743541110	0.805060420
	H	4.179116080	-1.997132650	3.204756950
	H	2.821739720	-0.780527670	4.905083580
	H	0.968538050	0.729178290	4.136222500
	H	1.690486270	-0.518943810	-5.221843620
	H	-0.036194370	-0.311112220	-4.880261590
	H	1.020962570	1.097312220	-4.990288800
	H	0.618009020	1.896951470	1.162763400
	Pd	-1.108364630	0.225853410	0.354093290
	O	-3.229305580	0.181814890	1.151918780
	C	-3.521163570	-0.365994480	0.056020240
	C	-4.923035210	-0.806220840	-0.274857670
	O	-2.592045960	-0.573423850	-0.819216990
	H	-5.610346920	-0.500318420	0.514363640
	H	-4.946597780	-1.894847200	-0.384904500
	H	-5.231611310	-0.374441520	-1.231298300
	Total DFT energy = -933.019972849024<br>One electron energy = -4393.738027331972<br>Coulomb energy = 2040.164476919358<br>Exchange-Corr. energy = -125.178495785542<br>Nuclear repulsion energy = 1545.732073349132			
3	C	1.120776490	-0.015203040	-1.497026560
	C	2.446372220	-0.408383270	-1.644897820
	C	2.709467040	-1.646783190	-2.231625820
	C	1.661963360	-2.483409070	-2.648364000
	C	0.352987060	-2.073890610	-2.476156590
	N	0.115743740	-0.862576650	-1.922175120
	C	0.469754890	1.178855420	-0.936498310
	C	-0.917757170	1.079989300	-1.257713140
	C	-1.156105200	-0.270683530	-1.595817630

	C	1.241176980	2.383771000	-0.479605970
	C	0.469336310	3.610178930	-0.059537750
	O	2.457936840	2.323740620	-0.466056530
	H	3.226066260	0.261541720	-1.307364320
	H	3.737616690	-1.965609450	-2.368173900
	H	1.859102120	-3.445451460	-3.105824550
	H	-0.511027700	-2.661242880	-2.763227990
	H	-1.666262220	1.858962030	-1.196642240
	H	-2.059317970	-0.732993070	-1.969284250
	H	1.172113390	4.361578340	0.300370540
	H	-0.090950320	4.022417400	-0.907226820
	H	-0.249500120	3.373145860	0.732284260
	Pd	-0.790283760	-0.015037070	0.577278360
	O	-0.313447320	0.046189600	2.604757100
	C	-1.257818710	-0.781080210	2.864424610
	C	-1.586165840	-1.203607740	4.254043170
	O	-1.919631500	-1.212112050	1.850929600
	H	-0.785567480	-0.915632670	4.936618420
	H	-1.748689340	-2.284021700	4.284363340
	H	-2.517891540	-0.717984690	4.563897860
	Total DFT energy =	-872.006968659128		
	One electron energy =	-3949.954754955727		
	Coulomb energy =	1818.257383733277		
	Exchange-Corr. energy =	-114.721854314071		
	Nuclear repulsion energy =	1374.412256877394		
4	N	-0.215679856	-1.489798583	-0.041262231
	C	-1.126365204	-2.474898174	-0.050081721
	C	-0.760776117	-3.815616995	-0.090532157
	C	0.599574968	-4.130665898	-0.123100815
	C	1.541802048	-3.107770192	-0.114047937
	C	1.119900987	-1.773264547	-0.072254803
	C	1.962611628	1.845808874	0.014617903
	C	1.256018605	0.643845514	-0.007371174
	C	1.964944506	-0.580490403	-0.055663970
	C	3.367685335	-0.585116311	-0.082223757
	C	4.062567031	0.621813166	-0.060468137
	C	3.361581699	1.831968825	-0.011979845
	H	-2.164014055	-2.158115210	-0.024018681
	H	-1.524400213	-4.584759669	-0.096596983
	H	0.922680172	-5.166769234	-0.155360746
	H	2.601455623	-3.334234506	-0.138604867
	H	1.423109232	2.787762263	0.052483131
	H	3.918759991	-1.521312093	-0.119978976
	H	5.148150767	0.621646581	-0.081339401
	H	3.909036465	2.770883626	0.005308086

	Pd -0.700926435 0.488833003 0.017842429<br>O -2.907432311 0.865450128 0.036111311<br>C -2.616338151 2.097123246 0.069765850<br>C -3.685174962 3.159129755 0.105716147<br>O -1.382486940 2.459725075 0.074754462<br>H -4.675523410 2.704393139 0.067765174<br>H -3.553909265 3.841609176 -0.739086359<br>H -3.583023433 3.750075112 1.021029880<br><br>Total DFT energy = -834.556592550964<br>One electron energy = -3747.570940083952<br>Coulomb energy = 1735.537245742924<br>Exchange-Corr. energy = -111.486631416948<br>Nuclear repulsion energy = 1288.963733207012
5	C -0.553029702 1.873053829 0.159595598<br>C -1.208795568 2.875308183 -0.569750750<br>C -1.728448473 2.666634808 -1.856601709<br>C -1.591128300 1.401757565 -2.435818911<br>C -0.940229171 0.381610241 -1.736503874<br>C -0.432441420 0.623875687 -0.463276330<br>C -0.017486397 2.150760387 1.543548121<br>C -0.751362371 -1.014675731 -2.316255809<br>C -2.401297133 3.795130903 -2.603907354<br>H -1.319854050 3.857093538 -0.112422458<br>H -1.993296765 1.208625755 -3.429287970<br>H -0.223895307 3.183067140 1.839617765<br>H -0.464122209 1.481233743 2.284634399<br>H 1.062861606 1.985081658 1.592400707<br>H -0.335055703 -1.772344837 -1.599938942<br>H -1.701412701 -1.460873278 -2.627711969<br>H -0.063446087 -1.022568093 -3.167368092<br>H -1.664283033 4.442442316 -3.095878997<br>H -2.987270923 4.428395518 -1.930286307<br>H -3.072658329 3.417052016 -3.380588952<br>Pd 0.372702436 -1.058988505 0.125753332<br>O 1.414211804 -2.759727881 1.197572353<br>C 1.593922620 -1.934371820 2.130099244<br>C 2.315374518 -2.296694719 3.401387205<br>O 1.150650736 -0.724928063 2.008009109<br>H 2.636491179 -3.338080937 3.366181572<br>H 1.653897770 -2.138660961 4.258313241<br>H 3.182466365 -1.642545966 3.531753142<br><br>Total DFT energy = -705.355170165722<br>One electron energy = -3004.492695650592<br>Coulomb energy = 1390.105675109587

	Exchange-Corr. energy = -94.851709755349<br>Nuclear repulsion energy = 1003.883560130632
6	C -2.944905099 -0.801838578 0.794034272<br>C -4.003727495 -1.690138527 0.932004645<br>C -3.879523615 -3.039079916 0.551000307<br>C -2.689524740 -3.531014623 0.021978095<br>C -1.620532351 -2.643005245 -0.119289686<br>C -1.742889508 -1.290701940 0.261678949<br>N -0.585825991 -0.561232267 0.050311444<br>S -0.006044828 -2.948569334 -0.745877661<br>C 0.407335558 -1.270988024 -0.465655823<br>C 1.647429849 -0.553496224 -0.706122691<br>C 2.855148594 -1.025556304 -1.235602735<br>C 1.558645231 0.817689500 -0.329850684<br>C 2.658334504 1.655826039 -0.491205707<br>C 3.848983949 1.150066101 -1.023214694<br>C 3.954072994 -0.191631746 -1.398049567<br>Cl 3.038533541 -2.748150647 -1.733144604<br>H -3.023855010 0.241330210 1.083070621<br>H -4.944990361 -1.337180742 1.341540898<br>H -4.724947983 -3.709474199 0.670859959<br>H -2.596649794 -4.571761647 -0.270891522<br>H 2.584098927 2.699644832 -0.202070492<br>H 4.708231962 1.803012193 -1.149868400<br>H 4.873676900 -0.589281465 -1.810752892<br>Pd -0.173967830 1.399300224 0.404175825<br>O -1.867585841 2.555082868 1.267861856<br>C -1.094063696 3.558670854 1.252623192<br>C -1.558551037 4.916604023 1.706996580<br>O 0.107895103 3.420765623 0.816753756<br>H -2.448576422 4.823277847 2.330709651<br>H -1.804794409 5.522766825 0.828453442<br>H -0.759745333 5.426403119 2.250245234<br><br>Total DFT energy = -1323.316594257154<br>One electron energy = -5596.401108171238<br>Coulomb energy = 2546.842361824607<br>Exchange-Corr. energy = -150.610293229781<br>Nuclear repulsion energy = 1876.852445319258
7	C 0.921424699 -0.851710359 -0.374906179<br>C 1.643342007 -1.981622321 -0.724112865<br>C 2.985139768 -1.852759088 -1.158313011<br>C 3.614048301 -0.619161882 -1.248814015<br>C 2.908975286 0.555207637 -0.901239709<br>C 1.569341702 0.403337227 -0.469943873<br>C 0.807928085 1.545888831 -0.106747640

	C	3.449447242	1.889136955	-0.953022031
	C	2.709130122	2.984687057	-0.601577481
	C	1.346132781	2.851107796	-0.160876350
	N	-0.472117564	1.290468573	0.294793797
	C	-1.256745377	2.305796991	0.652780089
	C	-0.801105932	3.635150196	0.628150699
	C	0.496178449	3.909361909	0.222917541
	H	1.187443689	-2.965896713	-0.667071791
	H	3.536596490	-2.749946252	-1.428240663
	H	4.644898176	-0.550213738	-1.585324390
	H	4.477290596	2.018262818	-1.282729461
	H	3.143345985	3.979296651	-0.650767152
	H	-2.264820998	2.051040639	0.963689186
	H	-1.474955531	4.429977546	0.927946544
	H	0.861551574	4.932619837	0.199085847
	Pd	-0.943877372	-0.716985059	0.261455678
	O	-3.036055721	-1.135222785	0.923503942
	C	-2.768670183	-2.356019881	0.721963400
	C	-3.780234405	-3.439552685	0.991082681
	O	-1.601296599	-2.688468185	0.294958154
	H	-4.772571289	-3.007396645	1.125317010
	H	-3.496950425	-3.978618987	1.901315122
	H	-3.784926355	-4.160098155	0.169377196
	Total DFT energy =	-910.785828638370		
	One electron energy =	-4286.683389188997		
	Coulomb energy =	1990.265481243157		
	Exchange-Corr. energy =	-122.380694558873		
	Nuclear repulsion energy =	1508.012773866344		
8	C	1.787571489	1.140436663	-1.897550661
	C	2.246580311	0.174798850	-2.791739983
	C	1.399765530	-0.318497768	-3.787879164
	C	0.092454589	0.159425859	-3.885765049
	C	-0.366690025	1.127454992	-2.991340508
	C	0.474933903	1.628417568	-1.989970198
	C	-0.020412723	2.669471357	-1.031194634
	S	-0.624983096	2.050091454	0.637041776
	C	-1.827022341	0.753563801	0.263571890
	C	-3.133511014	0.829182278	-0.207997711
	C	-1.207641379	-0.439603966	0.614113743
	C	-1.907037116	-1.637600434	0.479192187
	C	-3.228934954	-1.609573355	-0.000460435
	C	-3.822293254	-0.379378330	-0.341069559
	C	-4.022188584	-2.891117406	-0.116904187
	H	2.448508634	1.513308922	-1.119590095
	H	3.265841522	-0.191241096	-2.711858507

	H	1.758986657	-1.069956656	-4.484931704
	H	-0.570455350	-0.218365256	-4.658648794
	H	-1.385394399	1.497178806	-3.071326125
	H	0.768373038	3.371864883	-0.747103076
	H	-0.854009316	3.242539625	-1.444715380
	H	-3.601310370	1.775517995	-0.465541542
	H	-1.442764833	-2.585189614	0.739249821
	H	-4.844079487	-0.370782512	-0.711873472
	H	-4.768041585	-2.831170144	-0.915250735
	H	-3.371138058	-3.745890696	-0.321728454
	H	-4.558784475	-3.106752267	0.815558451
	Pd	0.608293005	0.046601900	1.193059318
	O	2.742271118	0.150577302	1.854301957
	C	2.686538487	-1.107959097	1.971558005
	C	3.874700429	-1.911042617	2.435972160
	O	1.594153342	-1.727142146	1.692539140
	H	4.728566285	-1.256851265	2.615502527
	H	4.130056445	-2.660940543	1.681387894
	H	3.617870548	-2.447056677	3.354603544
	Total DFT energy = -1295.302914717103<br>One electron energy = -5498.334435838654<br>Coulomb energy = 2502.619977254335<br>Exchange-Corr. energy = -148.468842627926<br>Nuclear repulsion energy = 1848.880386495142			
9	C	1.813607530	-2.200189880	-1.051155410
	C	1.170951140	-0.979629330	-1.206941980
	N	-0.053720090	-0.926591970	-1.838529220
	C	-0.679439030	-2.028620850	-2.315730790
	C	-0.059715080	-3.256592350	-2.185806640
	C	1.191219760	-3.342541480	-1.552647310
	C	-0.532745380	0.431179740	-1.810422290
	C	1.505171760	0.392539050	-0.811345090
	C	0.546964700	1.256420240	-1.429810070
	C	2.770337780	0.753322840	-0.269789500
	N	3.811708030	1.015135540	0.176015320
	H	2.777206840	-2.240100830	-0.556309890
	H	-1.643672140	-1.880537500	-2.786755260
	H	-0.548965980	-4.138832070	-2.580124830
	H	1.679261940	-4.306352650	-1.454784490
	H	-1.418501440	0.686517320	-2.374702220
	H	0.613554280	2.330594690	-1.541287930
	Pd	-0.441414040	0.714838720	0.395714640
	O	-0.409521060	0.751097830	2.472643960
	C	-1.676413900	0.942351140	2.503423420
	C	-2.422725290	1.155198250	3.771823890

	O	-2.262232820	0.956170100	1.358037880
	H	-1.782418600	0.939764170	4.627915790
	H	-3.313271770	0.521314050	3.787295680
	H	-2.756607090	2.197198840	3.818386520
	Total DFT energy =	-811.193021369581		
	One electron energy =	-3366.425576437523		
	Coulomb energy =	1547.477503449683		
	Exchange-Corr. energy =	-106.330579417900		
	Nuclear repulsion energy =	1114.085631036159		
10	N	-2.950914400	-0.649699220	0.020924690
	C	-3.061637510	-2.011087330	0.139192340
	C	-2.047126760	-2.760590860	-0.399001900
	S	-0.927608760	-1.743893480	-1.239566540
	C	-1.911508000	-0.307522300	-0.665871880
	C	-1.268502550	1.000887310	-0.728864370
	C	-1.714404990	2.049900400	0.116998120
	C	-0.141682790	1.214374770	-1.588597770
	C	0.518744600	2.464404480	-1.569399550
	C	0.077057690	3.467145400	-0.724812610
	C	-1.048985780	3.260420880	0.106033840
	H	-3.925303590	-2.430564770	0.642159750
	H	-1.949754120	-3.836962960	-0.432139830
	H	-2.568952000	1.874682900	0.760615300
	H	0.066359360	0.531915960	-2.409546430
	H	1.352936430	2.632884650	-2.241770030
	H	0.581136910	4.427937110	-0.712947280
	H	-1.391827660	4.064265910	0.749721630
	Pd	0.612978870	-0.289521060	0.047261590
	O	1.787970390	-1.425849220	1.316571570
	C	2.446441590	-0.378340280	1.654649560
	C	3.585224700	-0.420321070	2.608003090
	O	2.062627630	0.714486180	1.091138840
	H	3.535295780	-1.326778590	3.213488000
	H	3.575780410	0.470501780	3.240476330
	H	4.522925420	-0.423113870	2.040732840
	Total DFT energy =	-1155.637075612617		
	One electron energy =	-4358.364095572560		
	Coulomb energy =	1946.910568726199		
	Exchange-Corr. energy =	-125.636760062229		
	Nuclear repulsion energy =	1381.453211295973		
11	N	-1.024033930	-3.685910700	-0.346903440
	C	-2.218137880	-3.704491860	0.242983430
	C	-2.653524440	-2.725033720	1.163730830
	C	-1.805498840	-1.680355460	1.452574960

	N	-0.592677680	-1.665582860	0.841049420
	C	-0.218050280	-2.683309470	-0.032509350
	N	1.061431910	-2.497774810	-0.490677210
	C	0.494010500	-0.723210880	0.903106410
	C	1.502615870	-1.411529910	0.100492840
	H	-2.869506360	-4.534506720	-0.019286500
	H	-3.629112480	-2.786966490	1.629763470
	H	-2.035311790	-0.868748370	2.134208820
	H	0.707367240	-0.299593980	1.882996040
	H	2.514805400	-1.051698180	-0.050229150
	Pd	0.118034520	0.902767600	-0.369200340
	O	0.047709400	2.836992420	-1.216530690
	C	0.812395200	3.247301170	-0.289869130
	C	1.276508680	4.652010370	-0.151715650
	O	1.170985680	2.342739830	0.577453540
	H	1.107344480	5.188287860	-1.086970770
	H	2.334492770	4.674134670	0.121581440
	H	0.710852110	5.138721750	0.650640290
	Total DFT energy =	-751.399959035685		
	One electron energy =	-3015.372896705027		
	Coulomb energy =	1374.195903413361		
	Exchange-Corr. energy =	-96.714448665475		
	Nuclear repulsion energy =	986.491482921455		
12	C	-0.926757440	3.987274610	0.891859010
	C	-1.582916440	2.763591590	0.985003920
	C	-0.905588610	1.609669550	0.567744600
	C	0.417037390	1.650687270	0.058794250
	C	1.052795800	2.888342000	-0.025697090
	C	0.380283680	4.044778270	0.389467950
	C	-1.413531480	0.266149670	0.583315920
	N	-0.648736830	-0.731246310	0.147978300
	C	-1.391465040	-1.876916840	0.285130870
	C	-2.604347600	-1.529494270	0.807949730
	O	-2.609667560	-0.157582040	0.994723650
	C	-3.811317420	-2.269882220	1.172894760
	O	-4.821367690	-1.778526630	1.626482340
	O	-3.647026160	-3.593399180	0.933181980
	H	-1.427469740	4.896770840	1.208655220
	H	-2.596075460	2.696814370	1.371023770
	H	2.065729410	2.951188930	-0.412151860
	H	0.882464770	5.006452320	0.320874230
	H	-1.023549580	-2.852131440	0.007358580
	H	-4.480688800	-4.021496860	1.189359440
	Pd	1.208338850	-0.087379800	-0.470516930
	O	2.589788320	-1.679589330	-1.183771290

	C 3.427900580 -0.752969750 -1.380750060<br>C 4.798552760 -1.026309420 -1.939352910<br>O 3.099719690 0.461981840 -1.102698180<br>H 4.936036600 -2.095885690 -2.100635820<br>H 5.559250330 -0.650990750 -1.248569790<br>H 4.921476000 -0.489039450 -2.884603260<br><br>Total DFT energy = -1020.904411819450<br>One electron energy = -4494.346351043626<br>Coulomb energy = 2070.040913660952<br>Exchange-Corr. energy = -131.095333526886<br>Nuclear repulsion energy = 1534.496359090110

4.2 Literature test

No.	Cartesian coordinates, energies
1	['C', 2.30133782, 1.44348591, -0.03963458, conf1],<br>['C', 3.63911371, 1.56853988, -0.38985525, conf1],<br>['C', 4.28754305, 0.52465496, -1.06720957, conf1],<br>['C', 3.6492455, -0.65791611, -1.41624558, conf1],<br>['C', 2.3039187, -0.7760592, -1.06055282, conf1],<br>['C', 1.63114359, 0.25930172, -0.37958668, conf1],<br>['N', 0.30750096, -0.03756023, -0.1126917, conf1],<br>['S', 1.20979335, -2.12184474, -1.34597522, conf1],<br>['C', -0.0703119, -1.23140463, -0.54656903, conf1],<br>['C', -1.45253243, -1.59612716, -0.29350031, conf1],<br>['C', -2.1636537, -0.5707437, 0.39442964, conf1],<br>['C', -2.13148714, -2.77479024, -0.62898644, conf1],<br>['C', -3.46977631, -2.95973113, -0.30701958, conf1],<br>['C', -4.15189752, -1.94358166, 0.36764298, conf1],<br>['C', -3.50520444, -0.75359642, 0.71819695, conf1],<br>['Cl', -1.29201435, -4.11093753, -1.49878355, conf1],

	['Cl', 6.01540307, 0.7262328, -1.50113866, conf1],<br>['H', 1.77544226, 2.23552394, 0.48446683, conf1],<br>['H', 4.19004915, 2.46910267, -0.14386772, conf1],<br>['H', 4.17934177, -1.44511992, -1.93976345, conf1],<br>['H', -3.96604574, -3.88366632, -0.58092768, conf1],<br>['H', -5.1993865, -2.08733999, 0.61982559, conf1],<br>['H', -4.04332994, 0.03074856, 1.24224166, conf1],<br>['Pd', -1.13226175, 1.05145666, 0.82652535, conf1],<br>['O', -0.45180057, 3.04343045, 1.53584521, conf1],<br>['C', -1.62948941, 3.2087811, 1.97448317, conf1],<br>['C', -2.02909694, 4.46055334, 2.70976585, conf1],<br>['O', -2.49940034, 2.27670344, 1.80551826, conf1],<br>['H', -1.27310723, 5.23701856, 2.57985165, conf1],<br>['H', -2.13315191, 4.23381943, 3.77717406, conf1],<br>['H', -3.00239937, 4.8103102, 2.35356162, conf1]];<br><br>Total DFT energy = -1337.668163472253<br>One electron energy = -5797.271109806028<br>Coulomb energy = 2647.005077803946<br>Exchange-Corr. energy = -153.129626237135<br>Nuclear repulsion energy = 1965.518043390692
2	['C', -1.20127034, 3.90897974, 0.225632, conf1],<br>['C', -2.06617401, 2.8876962, -0.1668913, conf1],<br>['C', -1.6588337, 1.54800304, -0.11453308, conf1],<br>['C', -0.36526124, 1.2233689, 0.32861178, conf1],<br>['C', 0.49625978, 2.25479046, 0.72226983, conf1],<br>['C', 0.08096629, 3.58901667, 0.67300936, conf1],

	['C', -2.66087408, 0.47181243, -0.46910601, conf1],<br>['N', -2.10208637, -0.56191975, -1.34721579, conf1],<br>['N', -0.93769659, -1.15767336, -1.08920796, conf1],<br>['C', -0.80713478, -2.10764592, -2.02703656, conf1],<br>['C', -1.94539295, -2.0432876, -2.83448048, conf1],<br>['N', -2.7466105, -1.06743608, -2.38348073, conf1],<br>['H', -1.52721592, 4.94438751, 0.17927251, conf1],<br>['H', -3.06931391, 3.13049482, -0.51269147, conf1],<br>['H', 1.49172451, 2.01173935, 1.08232718, conf1],<br>['H', 0.76333719, 4.37634427, 0.9840973, conf1],<br>['H', -3.02949691, -0.0383832, 0.43042988, conf1],<br>['H', -3.51871019, 0.87221535, -1.01055529, conf1],<br>['H', 0.06477649, -2.7426098, -2.05540415, conf1],<br>['H', -2.2169853, -2.63122264, -3.69825025, conf1],<br>['Pd', 0.34495957, -0.62744055, 0.39651335, conf1],<br>['O', 1.61851686, -2.39421399, 0.93000998, conf1],<br>['C', 2.19052003, -1.6277235, 1.75774357, conf1],<br>['C', 3.30926937, -2.11155152, 2.64410006, conf1],<br>['O', 1.80811608, -0.40211505, 1.85681791, conf1],<br>['H', 3.56750194, -3.14417894, 2.40265232, conf1],<br>['H', 3.00029749, -2.0448494, 3.69299997, conf1],<br>['H', 4.18556825, -1.46699623, 2.52031527, conf1]<br><br>total DFT energy = -867.798334014867<br>One electron energy = -3905.501801747304<br>Coulomb energy = 1806.797996591963

	Exchange-Corr. energy = - 114.436644094880<br>Nuclear repulsion energy = 1344.4455669
3	['C', -1.7220706, -0.1288945, 1.26378046, conf1],<br>['C', -0.43822612, -0.13401945, 0.71110744, conf1],<br>['C', 0.65901354, 0.1447396, 1.53374165, conf1],<br>['C', 0.43807567, 0.41362376, 2.89306233, conf1],<br>['C', -0.84065962, 0.41719612, 3.45250601, conf1],<br>['C', -1.94689443, 0.14213704, 2.61935513, conf1],<br>['C', -3.35234515, 0.12674614, 3.17243412, conf1],<br>['C', -1.02616358, 0.70855535, 4.9232287, conf1],<br>['N', 2.01640731, 0.05555114, 1.07517879, conf1],<br>['C', 2.82190297, 0.84671851, 0.27882551, conf1],<br>['C', 2.23342265, 2.04881997, -0.50203492, conf1],<br>['O', 4.00891993, 0.55722041, 0.18043212, conf1],<br>['C', 1.53516967, 3.06203511, 0.4300035, conf1],<br>['C', 3.39932373, 2.75298047, -1.22405409, conf1],<br>['C', 1.26041504, 1.53271462, -1.58618574, conf1],<br>['H', -2.57324951, -0.36770213, 0.6305484, conf1],<br>['H', 1.30043435, 0.62314291, 3.52296463, conf1],<br>['H', -3.4633891, -0.6143807, 3.97469782, conf1],<br>['H', -4.0803484, -0.11507436, 2.39241875, conf1],<br>['H', -3.63072014, 1.09870852, 3.60063888, conf1],<br>['H', -1.50586312, -0.12964253, 5.44530816, conf1],<br>['H', -0.06630735, 0.89942075, 5.41274065, conf1],<br>['H', -1.66432979, 1.5868906, 5.08624521, conf1],<br>['H', 2.57870421, -0.66068413, 1.52427013, conf1],

	['H', 1.18954652, 3.92293691, -0.15489722, conf1],<br>['H', 2.23603788, 3.43408958, 1.18561237, conf1],<br>['H', 0.673013, 2.63599211, 0.94785828, conf1],<br>['H', 3.01916105, 3.6120593, -1.78907879, conf1],<br>['H', 4.14051847, 3.10922191, -0.5037932, conf1],<br>['H', 3.91202955, 2.07492442, -1.91085725, conf1],<br>['H', 0.99439616, 2.32125904, -2.2993357, conf1],<br>['H', 1.70186034, 0.7125516, -2.1626215, conf1],<br>['H', 0.26473607, 1.28570422, -1.11990599, conf1],<br>['Pd', -0.23580393, -0.54503323, -1.20789314, conf1],<br>['O', -0.42696519, -1.58990692, -3.18150564, conf1],<br>['C', -1.08983093, -2.46307319, -2.55646803, conf1],<br>['C', -1.64330658, -3.68850749, -3.23353481, conf1],<br>['O', -1.29567082, -2.31519105, -1.28932207, conf1],<br>['H', -1.50911565, -3.61460839, -4.31422666, conf1],<br>['H', -2.70416869, -3.80328978, -2.99062013, conf1],<br>['H', -1.12222287, -4.57718571, -2.86043416, conf1]];<br><br>Total DFT energy = -991.963771763584<br>One electron energy = -4971.255833761329<br>Coulomb energy = 2318.276526498318<br>Exchange-Corr. energy = -134.741739549086<br>Nuclear repulsion energy = 1795.757275048513
4	['C', -2.46492633, -1.73587212, -0.17761153, conf1],<br>['C', -3.22225775, -1.24800424, 0.85787209, conf1],<br>['C', -3.26033621, 0.14132733, 1.18538526, conf1],<br>['C', -2.49080285, 1.03411524, 0.48635834, conf1],<br>['C', -1.58844999, 0.56638137, -0.53830527, conf1],

	['C', -1.70801098, -0.82168768, -0.97951062, conf1],<br>['N', -1.19866561, -1.17448409, -2.17453049, conf1],<br>['C', -1.2342277, 1.42347646, -1.7410749, conf1],<br>['S', -0.41854873, 0.16362955, -2.818778, conf1],<br>['C', 1.2489932, 0.2537623, -2.1188282, conf1],<br>['C', 1.47721073, 0.29526987, -0.74257531, conf1],<br>['C', 2.27286517, 0.28744247, -3.07605708, conf1],<br>['C', 3.59302593, 0.38784862, -2.64665206, conf1],<br>['C', 3.85847482, 0.44706656, -1.27569719, conf1],<br>['C', 2.82198925, 0.40085545, -0.33936248, conf1],<br>['H', -2.48043754, -2.78361979, -0.45786571, conf1],<br>['H', -3.82370677, -1.93789192, 1.44411343, conf1],['H', -3.88247789, 0.47828918, 2.00789579, conf1],['H', -2.51290292, 2.09382713, 0.72575244, conf1],['H', -2.1274945, 1.7746727, -2.27374277, conf1],['H', -0.54875671, 2.25311089, -1.56817957, conf1],['H', 2.03430008, 0.24089217, -4.13609866, conf1],['H', 4.40230919, 0.41788929, -3.37038503, conf1],['H', 4.88586294, 0.52301459, -0.9274796, conf1],['H', 3.05591618, 0.42911534, 0.72004928, conf1],['Pd', 0.14917222, 0.14171261, 0.73683999, conf1],['O', -0.61453545, -0.19890851, 2.81446689, conf1],['C', 0.58768574, -0.2909001, 3.20200246, conf1],['C', 0.92688822, -0.5549313, 4.64875901, conf1],['O', 1.53921054, -0.16122368, 2.3540076, conf1],['H', 0.01869591, -0.6121226, 5.25192276, conf1],['H', 1.57542108, 0.24204341, 5.02777993, conf1],['H', 1.48344808, -1.49489703, 4.72989558, conf1]];<br><br>Total DFT energy = -1310.068341737213<br>One electron energy = -5634.944500191906<br>Coulomb energy = 2566.540394617571<br>Exchange-Corr. energy = -148.831612646038<br>Nuclear repulsion energy = 1905.642309180845

5	['N', -0.46972902, -0.22338131, -0.09497911, conf1],['C', -0.27739137, 0.36147258, 1.11510552, conf1],['C', -0.88570032, 1.62474959, 1.31585105, conf1],['C', -1.62619965, 2.15309035, 0.26346321, conf1],['N', -1.82228974, 1.58247696, -0.94207082, conf1],['C', -1.22245157, 0.41197769, -1.04510232, conf1],['N', -0.88601725, 2.46691979, 2.40541693, conf1],['C', -1.61625841, 3.48651659, 2.00852901, conf1],['N', -2.10327796, 3.35876754, 0.72389918, conf1],['C', -2.91275398, 4.32052202, -0.03681303, conf1],['C', -2.08868796, 5.3898243, -0.727407, conf1],['N', 0.44776643, -0.20068799, 2.09398649, conf1],['C', 1.14054275, -1.43216592, 2.12505439, conf1],['C', 2.5637327, -3.82679991, 2.36328021, conf1],['C', 2.5247099, -2.92752649, 3.44041743, conf1],['C', 1.81864611, -1.74319981, 3.31740735, conf1],['C', 1.17045137, -2.31948301, 1.0424652, conf1],['C', 1.88952975, -3.5179159, 1.17749207, conf1],['O', 3.28340239, -4.96950877, 2.57215299, conf1],['C', 3.35765271, -5.91377403, 1.51618733, conf1],['C', -1.33701409, 5.07373019, -1.86785779, conf1],['C', -0.56961641, 6.0532162, -2.49710473, conf1],['C', -0.54894214, 7.35865906, -1.99785909, conf1],['C', -1.29955333, 7.68118316, -0.86677499, conf1],['C', -2.0654883, 6.69889624, -0.23483085, conf1],['H', -1.31814998, -0.13286917, -1.97880995, conf1],['H', -1.8407319, 4.35972398, 2.60718324, conf1],['H', -3.62810798, 4.77126525, 0.65755404, conf1],['H', -3.47605629, 3.73047467, -0.76479053, conf1],['H', 0.49525921, 0.36397566, 2.93468697, conf1],['H', 3.05125809, -3.17693423, 4.35568809, conf1],['H', 1.78868672, -1.04572598, 4.15313196, conf1],['H', 1.90938767, -4.20053273, 0.33632895, conf1],['H', 3.97154587, -6.73494719, 1.89188053, conf1],['H', 2.36466699, -6.29590385, 1.24522381, conf1],['H', 3.83047833, -5.48475009, 0.62297585, conf1],['H', -1.35673243, 4.05862539, -2.25673161, conf1],['H', 0.00913144, 5.79910393, -3.38088649, conf1],['H', 0.04734848, 8.12113942,

	-2.49151331, conf1],['H', -1.29167819, 8.69512996, -0.47624731, conf1],['H', -2.65365585, 6.95560541, 0.64397189, conf1],['Pd', 0.27401003, -2.02492113, -0.68570712, conf1],['O', -0.44961723, -2.32345516, -2.78072908, conf1],['C', 0.16426007, -3.43140521, -2.76215363, conf1],['C', 0.13249913, -4.36526242, -3.94566602, conf1],['O', 0.81010452, -3.78151083, -1.70919596, conf1],['H', -0.23163921, -3.84388732, -4.83314774, conf1],['H', -0.53728147, -5.20484824, -3.72570435, conf1],['H', 1.12933021, -4.77695753, -4.12817599, conf1]<br><br>Total DFT energy = -1438.483434407151<br>One electron energy = -7873.011443280018<br>Coulomb energy = 3680.188553568017<br>Exchange-Corr. energy = -194.315164224758<br>Nuclear repulsion energy = 2947.235264116937
6	['C', -0.24896286, -1.01395028, 3.58267421, conf1],['C', -0.59265438, -0.60774369, 2.26535982, conf1],['C', -1.95310196, -0.24204769, 1.99799875, conf1],['C', -2.90794984, -0.27983399, 3.05036105, conf1],['C', -2.5375359, -0.6766206, 4.31530025, conf1],['C', -1.19685698, -1.04985202, 4.58137761, conf1],['C', 0.37257367, -0.55422698, 1.22004302, conf1],['C', 0.01475613, -0.1412581, -0.0384571, conf1],['C', -1.34380342, 0.2151119, -0.32463543, conf1],['C', -2.31648279, 0.15610208, 0.67834543, conf1],['C', -1.6541131, 0.6748025, -1.67847294, conf1],['O', -2.68555851, 0.96883882, -2.227948, conf1],['O', -0.45131642, 0.76016996, -2.41753397, conf1],['O', -3.61225524, 0.52899451, 0.46165776, conf1],['C', -4.45801516, -0.44423048, -0.16736031, conf1],['H', 0.78111903, -1.29329774, 3.78825799, conf1],['H', -3.92652244, 0.02410071, 2.83631379, conf1],['H', -3.27196881, -0.70082632, 5.11537797, conf1],['H', -0.91601839, -1.36171885, 5.58385691, conf1],['H', 1.40076072, -0.83463173, 1.43229373, conf1],['H', -0.64868636,

	1.00473954, -3.34050659, conf1],['H', -<br>5.44947308, 0.00999008, -0.21245503,<br>conf1],['H', -4.11699777, -0.6675389, -<br>1.18128177, conf1],['H', -4.49932557, -<br>1.36207621, 0.43226577, conf1],['Pd',<br>1.30381414, 0.00689531, -1.5126726, conf1],['O',<br>3.18381822, -0.0805382, -2.73294128,<br>conf1],['C', 3.72276077, -0.56050001, -<br>1.69829768, conf1],['C', 5.17496173, -<br>0.95520772, -1.65590113, conf1],['O',<br>3.00654809, -0.72491328, -0.63534649,<br>conf1],['H', 5.65746755, -0.72230649, -<br>2.60669252, conf1],['H', 5.25875644, -<br>2.02799644, -1.45134271, conf1],['H',<br>5.67919223, -0.42574339, -0.84091608, conf1]<br><br>Total DFT energy = -1044.158088294759<br>One electron energy = -4834.841126912455<br>Coulomb energy = 2237.255996027257<br>Exchange-Corr. energy = -136.184545343106<br>Nuclear repulsion energy =<br>1688.779213376769

4.3 New reactions predictions

No.	Cartesian coordinates, energies
1	C 0.859175700 -2.574717800 -<br>1.497186220<br>C 0.325734300 -3.853633640 -<br>1.599078790<br>C -1.003243520 -4.139960970 -<br>1.218970590<br>C -1.849216100 -3.154238730 -<br>0.725913930<br>C -1.315204040 -1.866932380 -<br>0.628507240<br>C 0.013413990 -1.576233290 -<br>0.999834670<br>N 0.221041120 -0.216560140 -<br>0.757008390<br>N -1.868558910 -0.668266110 -<br>0.192859780<br>C -0.923625640 0.286681610 -<br>0.278320080<br>C -1.094989540 1.748205580<br>0.021223090

	S -2.407331190 2.192520690 1.235844930
	C 0.258568430 2.398359300 0.338855250
	C -2.129282520 1.093964080 2.684672700
	C -3.241665990 0.056494830 2.825082770
	O -3.656760950 -0.028558520 4.079870060
	O -3.681599300 -0.618344060 1.906907310
	C -4.695035500 -1.003793530 4.357425830
	H 1.879274380 -2.356712210 -1.788907180
	H 0.947128420 -4.656332130 -1.982270770
	H -1.373488450 -5.155004230 -1.317889340
	H -2.871932020 -3.368663290 -0.435701540
	H -2.746456030 -0.567133390 0.338442160
	H -1.497870490 2.225444920 -0.882428830
	H 0.162928410 3.470130900 0.532138590
	H 0.901824270 2.435762510 -0.601764170
	H 0.764677570 1.948473360 1.196248310
	H -2.078215450 1.709705710 3.582970560
	H -1.175200640 0.567834310 2.580577350
	H -4.899008200 -0.907527960 5.421794210
	H -4.338330330 -2.006874220 4.117123910
	H -5.583610320 -0.777441250 3.765955860
	Pd 1.897335340 0.889641160 -0.910799720
	O 3.314107910 -0.350077590 -1.699190450

	C 4.186888440 0.599847960 -<br>1.661580510<br>C 5.573798260 0.417618970 -<br>2.161036240<br>O 3.756059890 1.708244010 -<br>1.185286720<br>H 5.852491510 -0.637185520 -<br>2.131469680<br>H 6.263696250 1.019969350 -<br>1.565994950<br>H 5.623902120 0.765510270 -<br>3.199205710<br><br>Total DFT energy = -1479.446622737764<br>One electron energy = -6374.542679658208<br>Coulomb energy = 2890.662380886692<br>Exchange-Corr. energy = -<br>168.751556455525<br>Nuclear repulsion energy =<br>2173.185232489278
2	C -1.361539020 0.875830480 -<br>0.247062000<br>C -2.130847620 -0.039354000<br>0.700702410<br>C -1.380776720 -1.364517810<br>0.984776510<br>C -1.046598840 -2.168118810 -<br>0.292260500<br>C -0.452503230 -1.260465300 -<br>1.292547890<br>C -0.546035240 0.142335380 -<br>1.274058250<br>C 0.110130420 0.606979000 -<br>2.468724290<br>O 0.200275790 -1.691142120 -<br>2.391726320<br>C 0.681810400 -0.540097720 -<br>3.024206890<br>O -1.402828180 2.087785840 -<br>0.253564300<br>C -2.555962680 0.695895310<br>1.975297130<br>C -3.712028400 -0.013876450<br>2.662912820<br>O -4.037384330 0.596811760<br>3.804994900

	O -4.266207670 -1.002850700<br>2.222748140<br>C -5.142578110 0.022416790<br>4.539528570<br>H -3.053686860 -0.306472350<br>0.158606370<br>H -2.014133290 -1.978849850<br>1.626330920<br>H -0.453115350 -1.157232180<br>1.532779490<br>H -0.386318510 -3.019354710 -<br>0.095750080<br>H -1.964158250 -2.586826530 -<br>0.736349790<br>H 0.142086040 1.616015660 -<br>2.857988480<br>H 1.228322770 -0.709970920 -<br>3.941062060<br>H -2.864343170 1.717932470<br>1.733434790<br>H -1.723245200 0.793168040<br>2.681505420<br>H -6.050776570 0.044519860<br>3.933996300<br>H -4.917815620 -1.009877460<br>4.815442320<br>H -5.254392470 0.644056680<br>5.426187440<br>Pd 1.787351550 0.151611820 -<br>1.130745820<br>O 2.943801000 0.259894560<br>0.572043330<br>C 3.980103780 0.530223650 -<br>0.134730010<br>C 5.308052200 0.832480770<br>0.453405380<br>O 3.773724720 0.539037420 -<br>1.407087980<br>H 5.324750410 0.552984100<br>1.507972380<br>H 5.503059400 1.906691540<br>0.359557790<br>H 6.087641740 0.301865910 -<br>0.099790970<br><br>Total DFT energy = -1082.816402106921

	One electron energy = -5036.751017741624<br>Coulomb energy = 2318.823847388187<br>Exchange-Corr. energy = -<br>140.155232576514<br>Nuclear repulsion energy =<br>1775.266000823030
3	C -1.073381780 0.068021510 -<br>0.220112990<br>N -2.347800100 0.181121360 -<br>0.444047370<br>C -3.140415500 1.083589970<br>0.191639280<br>C -2.578869440 1.924743670<br>1.125790900<br>C -1.185873260 1.839849250<br>1.411028000<br>C -0.389202160 0.874973030<br>0.715141730<br>C 0.985962060 0.807648340<br>1.009989590<br>N -0.645267280 2.676401520<br>2.335775790<br>C 0.649574670 2.608316250<br>2.609660850<br>C 1.523232940 1.664104260<br>1.952419540<br>C 1.137719120 3.581539640<br>3.649470930<br>C 2.981909350 1.597320450<br>2.269501460<br>O 3.563529090 2.297910680<br>3.074946680<br>O 3.608556560 0.645042520<br>1.543312740<br>C 5.018759910 0.513477880<br>1.786835640<br>H -4.192888840 1.099824250 -<br>0.068281050<br>H -3.175008740 2.657920020<br>1.655860930<br>H 1.611278970 0.086539530<br>0.497112910<br>H 1.592836330 3.060972850<br>4.496420300<br>H 0.289561320 4.177774420<br>3.987203640

	H 1.918890200 4.232184230<br>3.247209380<br>H 5.205087750 0.255965170<br>2.832236740<br>H 5.354755150 -0.286468910<br>1.127847210<br>H 5.536758240 1.447246110<br>1.555195700<br>Pd -1.320633580 -1.287670000 -<br>1.570756850<br>O -1.731987650 -2.803594700 -<br>3.116570080<br>C -0.502910050 -3.100508130 -<br>3.070438970<br>C 0.082578360 -4.193795700 -<br>3.923532120<br>O 0.264059220 -2.457276990 -<br>2.263222190<br>H -0.656499240 -4.550015330 -<br>4.641843190<br>H 0.399050860 -5.023361160 -<br>3.283230490<br>H 0.969965460 -3.824035920 -<br>4.444251040<br><br>Total DFT energy = -1040.334639275808<br>One electron energy = -4689.221434419004<br>Coulomb energy = 2164.567828529027<br>Exchange-Corr. energy = -<br>135.971068541066<br>Nuclear repulsion energy =<br>1620.290035155235
4	C -0.097352870 0.109624890 -<br>1.731640360<br>C -0.694118870 1.215061480 -<br>1.005063810<br>C -0.075291090 2.426422000 -<br>1.521789030<br>C 0.850827620 2.013971100 -<br>2.506042010<br>N 0.802173870 0.603618380 -<br>2.594122670<br>C -2.013171680 -0.445664900<br>0.253291220<br>C -1.597523470 0.923395630 -<br>0.014056490

	C -0.464109730 -1.247216550 -1.474825520
	C -1.573163080 -1.479903860 -0.616704440
	C -0.219511490 3.787909480 -1.245416000
	C 0.563760390 4.697383020 -1.958825690
	C 1.474480850 4.262824130 -2.932828080
	C 1.634655360 2.904869100 -3.226188220
	C -3.116098380 -0.652929120 1.275306720
	C -3.406472310 -2.098511120 1.683650030
	H 1.364113430 0.048998910 -3.227404520
	H -2.020560080 1.711417180 0.603219760
	H -0.064776580 -2.049673900 -2.085832530
	H -1.997349530 -2.474244100 -0.548319520
	H -0.923721040 4.136674810 -0.496769420
	H 0.465803060 5.758887950 -1.758001060
	H 2.068808140 4.993253730 -3.471691330
	H 2.339860810 2.571272610 -3.980425110
	H -2.864835730 -0.061018190 2.163387110
	H -4.027920730 -0.195997280 0.864155170
	H -4.178213340 -2.112652020 2.456898610
	H -2.516444600 -2.586532110 2.093894160
	H -3.779244150 -2.698449630 0.847538320
	Pd 0.132108900 -1.213141280 0.729084380
	O 0.578581430 -1.291471800 2.757335590

	C 1.781942870 -1.651602430 2.495420180<br>C 2.756285460 -1.993811100 3.569169550<br>O 2.084357360 -1.725546740 1.251667680<br>H 2.464016040 -1.522754510 4.509076590<br>H 3.760902950 -1.682356260 3.275039630<br>H 2.764229600 -3.080719230 3.707607540<br><br>Total DFT energy = -951.658713276782<br>One electron energy = -4689.511750449346<br>Coulomb energy = 2173.743715291785<br>Exchange-Corr. energy = -129.430308302289<br>Nuclear repulsion energy = 1693.539630183068
5	C 0.254879100 -1.457562110 -0.037564740<br>C -0.550836070 -0.353862540 -0.022945440<br>C -1.953707170 -0.529325240 -0.030191270<br>C -2.501088370 -1.806958820 -0.049100780<br>C -1.622806670 -2.905838130 -0.061329070<br>C -0.204706220 -2.771752590 -0.056781850<br>C 0.289197000 -4.109033420 -0.075945920<br>N -1.844629750 -4.257312700 -0.080972710<br>N -0.701456990 -4.984310390 -0.090024310<br>Cl 2.048525070 -0.974151380 -0.044898380<br>H -2.599806520 0.342667890 -0.017821040<br>H -3.577176170 -1.951214640 -0.054340660<br>H 1.314809470 -4.448990770 -0.079592730

	H -2.726619140 -4.741827660 -<br>0.089796850<br>Pd 0.572385850 1.247504130<br>0.002594550<br>O 1.332754670 3.356490830<br>0.109294280<br>C 0.119818210 3.693039460<br>0.133331730<br>C -0.321940620 5.129011120<br>0.194279220<br>O -0.789576640 2.771324680<br>0.092899470<br>H 0.547401610 5.785121770<br>0.244041910<br>H -0.959097280 5.283575640<br>1.069881950<br>H -0.917922740 5.366410550 -<br>0.691987150<br><br>Total DFT energy = -749.352928108988<br>One electron energy = -3110.007622738734<br>Coulomb energy = 1434.629780425167<br>Exchange-Corr. energy = -98.071499036013<br>Nuclear repulsion energy =<br>1024.096413240591
6	C -1.035873140 0.039488670<br>0.555079170<br>C -1.740511770 0.551511510<br>1.635007160<br>C -1.067533430 1.390365910<br>2.540609740<br>C 0.272696550 1.753042520<br>2.435982880<br>C 0.968664950 1.232323620<br>1.347241620<br>C 0.331167250 0.394145410<br>0.426225750<br>C 1.031587310 -0.146325480 -<br>0.695972970<br>O 2.293595000 1.548597310<br>1.181638140<br>C 2.962122650 1.040707010<br>0.122768250<br>C 2.418761620 0.220370270 -<br>0.818139090

	F -1.761161360 1.879127410 3.581773420<br>O 0.409541200 -0.901130480 -1.504597570<br>C 4.403851890 1.452593340 0.041059380<br>O 4.761447370 2.256094220 1.047930540<br>O 5.133294100 1.079037710 -0.852439360<br>C 6.136845680 2.687978720 1.026206130<br>H -2.788498020 0.325554940 1.800857430<br>H 0.739802290 2.403579820 3.164153750<br>H 3.039415540 -0.134181880 -1.630874390<br>H 6.345734270 3.242438440 0.108583950<br>H 6.253123240 3.327011970 1.900167400<br>H 6.805675210 1.826247510 1.083137620<br>Pd -1.597236660 -1.143957110 -0.907577010<br>O -2.750724860 -2.461669000 -2.281672780<br>C -3.730621860 -2.259563010 -1.512537580<br>C -5.076283550 -2.890121860 -1.743064370<br>O -3.569446230 -1.495921330 -0.481845460<br>H -5.112384120 -3.347546550 -2.732344470<br>H -5.863546270 -2.138803750 -1.640652740<br>H -5.251121440 -3.657538960 -0.981938550<br><br>Total DFT energy = -1179.301553709704<br>One electron energy = -5236.766564466325<br>Coulomb energy = 2409.543133165935<br>Exchange-Corr. energy = -148.672461073728

	Nuclear repulsion energy = 1796.594338664414
7	N -3.518801540 -0.226613770 -<br>1.123711850<br>C -4.252034880 -0.493165880<br>0.026571610<br>C -5.555612020 -0.232360030 -<br>0.297904490<br>N -5.659987800 0.203673960 -<br>1.604083880<br>C -4.435201000 0.192431190 -<br>2.068752330<br>C -2.118357570 -0.330282380 -<br>1.286437900<br>C 0.108109950 -0.200972340 -<br>0.358143120<br>C -1.269497670 -0.092419270 -<br>0.194287620<br>C -1.594908910 -0.673516710 -<br>2.548561750<br>C -0.221833640 -0.766042380 -<br>2.723219630<br>C 0.625627800 -0.533236370 -<br>1.630931590<br>C 2.076224860 -0.603337910 -<br>1.648492630<br>O 2.733523890 -0.371823360 -<br>0.606121470<br>O 2.678654920 -0.916654120 -<br>2.784924390<br>C 4.122347840 -0.976577540 -<br>2.763730550<br>H -3.786323940 -0.875763660<br>0.920441830<br>H -6.428032900 -0.336650790<br>0.332032400<br>H -4.128701050 0.512248440 -<br>3.054464720<br>H -1.680669060 0.197418150<br>0.766875860<br>H -2.267262120 -0.893562570 -<br>3.369801850<br>H 0.194140350 -1.032202400 -<br>3.689976870<br>H 4.458086400 -1.733937240 -<br>2.052716590

	H 4.412104970 -1.242561560 -<br>3.778994970<br>H 4.536084620 -0.006080990 -<br>2.482959450<br>Pd 1.465333430 0.083881870<br>1.023627830<br>O 2.478551210 0.496773480<br>2.987918400<br>C 1.313343380 0.620742870<br>3.451544540<br>C 1.051058070 0.949037860<br>4.896167130<br>O 0.301106800 0.461553600<br>2.660251310<br>H 1.993059850 1.066582910<br>5.432397170<br>H 0.459153420 0.150440260<br>5.353377380<br>H 0.464951600 1.870322070<br>4.963342430<br><br>Total DFT energy = -1040.333081732739<br>One electron energy = -4750.732148288092<br>Coulomb energy = 2195.300602758361<br>Exchange-Corr. energy = -<br>135.983710832294<br>Nuclear repulsion energy =<br>1651.082174629286
8	C 2.584779520 -1.065563600 -<br>0.362058430<br>C 3.911066940 -1.082098640 -<br>0.740592480<br>C 4.612102460 0.084378550 -<br>1.127418150<br>C 3.909035040 1.287877030 -<br>1.115989540<br>C 2.567465770 1.321922230 -<br>0.734711630<br>C 1.883946240 0.153646920 -<br>0.354184690<br>C 0.488022330 0.261224350<br>0.037269760<br>O 1.951281010 2.549968840 -<br>0.751833540<br>C 0.659150340 2.642537570 -<br>0.394594370

	C -0.088309180 1.578707700 -<br>0.009175000<br>Cl 4.772703670 -2.652692760 -<br>0.735136210<br>C 6.059231180 0.043997320 -<br>1.535071410<br>O -0.212557640 -0.717986580<br>0.400691340<br>C -1.546030850 1.668241130<br>0.398047950<br>O -2.139134910 2.720244480<br>0.397206870<br>H 2.067002830 -1.971654060 -<br>0.069858640<br>H 4.395607530 2.213309030 -<br>1.404619690<br>H 0.279159300 3.655961480 -<br>0.450934860<br>H 6.686423250 -0.310391790 -<br>0.710308020<br>H 6.407599080 1.033854240 -<br>1.836708110<br>H 6.211605050 -0.649946080 -<br>2.367613830<br>Pd -2.195767610 -0.106242580<br>0.866244250<br>O -3.513168810 -1.848218270<br>1.488268410<br>C -4.421976440 -0.985123900<br>1.627998120<br>C -5.810085910 -1.356065720<br>2.083440480<br>O -4.172004040 0.255142820<br>1.379500040<br>H -5.887642010 -2.435249660<br>2.220243920<br>H -6.542367380 -1.018768280<br>1.344126190<br>H -6.037006330 -0.844239930<br>3.023491300<br><br>Total DFT energy = -1019.208176852926<br>One electron energy = -4621.786047057436<br>Coulomb energy = 2137.097544822834<br>Exchange-Corr. energy = -<br>132.481687397838

	Nuclear repulsion energy = 1597.962012779514
9	C 0.252185210 -2.172224420 0.336474690<br>C 0.748163090 -3.431650780 0.604854150<br>C 2.145547620 -3.676063730 0.569881380<br>C 3.013384420 -2.653341280 0.275154080<br>C 2.541179380 -1.340264050 0.002059190<br>C 1.130819830 -1.094302940 0.031247520<br>C 0.659699620 0.231096240 -0.251963130<br>N 3.461085600 -0.372355230 -0.285951450<br>C 2.992761670 0.832148110 -0.528420570<br>C 1.617227740 1.207163330 -0.501901590<br>O -0.021539030 -4.525426380 0.920965980<br>C -1.423194780 -4.333224750 0.985679280<br>C 1.357216590 2.663242100 -0.796049380<br>O 2.096172790 3.298572480 -1.554212900<br>N 0.308561540 3.236052150 -0.151313080<br>H -0.808264110 -1.959544070 0.368812820<br>H 2.498379460 -4.681436080 0.782795450<br>H 4.087300780 -2.813690220 0.242092590<br>H 3.716432970 1.608668510 -0.770499780<br>H -1.848962860 -5.301361000 1.262252160<br>H -1.695415380 -3.583466250 1.740447180<br>H -1.834244610 -4.018203590 0.016642210

	H 0.186083510 4.227066600 -<br>0.307699090<br>H -0.174336780 2.801969270<br>0.642602630<br>Pd -1.276100850 0.552746160 -<br>0.459135660<br>O -1.324809210 0.820091740 -<br>2.552268800<br>C -2.588246300 0.994466960 -<br>2.580355090<br>O -3.310143500 0.946759700 -<br>1.554978370<br>O -1.628941950 0.194509500<br>1.524925730<br>C -1.433565440 1.118266320<br>2.400204850<br>O -0.957076780 2.247276580<br>2.256898170<br>H -1.762024560 0.798249630<br>3.412868960<br>H -3.043945350 1.195739400 -<br>3.568389250<br><br>Total DFT energy = -1190.262614463902<br>One electron energy = -5721.460522287092<br>Coulomb energy = 2662.404926802650<br>Exchange-Corr. energy = -<br>153.055256733222<br>Nuclear repulsion energy =<br>2021.848237753763

5. References

[i] J.-D. Chai and M. Head-Gordon, "Long-range corrected hybrid density functionals with damped atom-atom dispersion corrections," Phys. Chem. Chem. Phys., 10 (2008) 6615-20.

[ii] A. D. Becke, "Density-functional thermochemistry. III. The role of exact exchange," J. Chem. Phys., 98 (1993) 5648-52.

[iii] J. M. Tao, J. P. Perdew, V. N. Staroverov, and G. E. Scuseria, "Climbing the density functional ladder: Nonempirical meta-generalized gradient approximation designed for molecules and solids," Phys. Rev. Lett., 91 (2003) 146401.

[iv] Y. Zhao and D. G. Truhlar, "The M06 suite of density functionals for main group thermochemistry, thermochemical kinetics, noncovalent interactions, excited states, and transition elements: two new functionals and systematic testing of four M06-class functionals and 12 other functionals," Theor. Chem. Acc., 120 (2008) 215-41.

[v] Andersson, M. P., & Uvdal, P. (2005). New scale factors for harmonic vibrational frequencies using the B3LYP density functional method with the triple-ζ basis set 6-311+ G (d, p). The Journal of Physical Chemistry A, 109(12), 2937-2941.

[vi] Valiev, M., Bylaska, E. J., Govind, N., Kowalski, K., Straatsma, T. P., Van Dam, H. J., ... & De Jong, W. A. (2010). NWChem: a comprehensive and scalable open-source solution for large scale molecular simulations. Computer Physics Communications, 181(9), 1477-1489.
[vii] Krishnan, R. B. J. S., Binkley, J. S., Seeger, R., & Pople, J. A. (1980). Self-consistent molecular orbital methods.
[viii] Van Speybroeck, V., Van Neck, D., Waroquier, M., Wauters, S., Saeys, M., & Marin, G. B. (2000). Ab initio study of radical addition reactions: Addition of a primary ethylbenzene radical to ethene (I). The Journal of Physical Chemistry A, 104(46), 10939-10950.
[ix] Wang, J., Nie, Z., Li, Y., Tan, S., Jiang, J., Jiang, P., & Ding, Q. (2013). Pd-catalysed ortho-CH acylation/cross coupling of 2-arylbenzo [d] thiazoles with aldehydes using tert-butyl hydroperoxide as oxidant. Journal of Chemical Research, 37(5), 263-267.
[x] Tian, Q., He, P., & Kuang, C. (2014). Palladium-catalyzed ortho-acylation of 2-benzyl-1, 2, 3-triazoles with aldehydes. Organic & biomolecular chemistry, 12(38), 7474-7477.
[xi] Chan, C. W., Zhou, Z., & Yu, W. Y. (2011). Palladium (II)-Catalyzed Direct ortho-C□ H Acylation of Anilides by Oxidative Cross-Coupling with Aldehydes using tert-Butyl Hydroperoxide as Oxidant. Advanced synthesis & catalysis, 353(16), 2999-3006.
[xii] Banerjee, A., Santra, S. K., Guin, S., Rout, S. K., & Patel, B. K. (2013). Palladium-Catalyzed ortho-Aroylation of 2-Arylbenzothiazoles and 2-Arylbenzoxazoles with Aldehydes. European Journal of Organic Chemistry, 2013(7), 1367-1376.
[xiii] Allu, S., & Swamy, K. K. (2015). Palladium-catalysed ortho-acylation of 6-anilinopurines/purine nucleosides via C–H activation. RSC Advances, 5(112), 92045-92054.
[xiv] Giri, R., & Yu, J. Q. (2008). Synthesis of 1, 2-and 1, 3-dicarboxylic acids via Pd (II)-catalyzed carboxylation of aryl and vinyl C− H bonds. Journal of the American Chemical Society, 130(43), 14082-14083.
